# Patient navigation in neuro-oncology: A scoping review of programs supporting patients with CNS tumors

**DOI:** 10.1016/j.neuros.2026.100067

**Published:** 2026-08-24

**Authors:** Steven Shafeek, Kevin Placide, Cleopatra Elshiekh, Anna Druker, Liana Wu, Antonuos Ishak, Johanna Goldberg, Francesca M. Gany, Joshua A. Budhu

**Affiliations:** a CUNY School of Medicine, City University of New York, New York, NY, USA; b Immigrant and Cancer Disparities Service, Department of Psychiatry, Memorial Sloan Kettering Cancer Center, New York, NY, USA; c University of Maryland, College Park, Maryland, USA; d Department of Anthropology, Case Western Reserve University, Cleveland, Ohio, USA; e MSKCC Library, Memorial Sloan Kettering Cancer Center, New York, NY, USA; f Department of Neurology, Memorial Sloan Kettering Cancer Center, New York, NY, USA

**Keywords:** Patient navigation, CNS tumors, Glioblastoma, Health equity, Patient-reported outcomes

## Abstract

**Purpose of Review:**

Patients with primary central nervous system (CNS) tumors and brain metastases face substantial barriers across the cancer care continuum, including cognitive impairment, complex care transitions, psychosocial distress, and financial hardship. Patient navigation programs aim to support patients and caregivers by facilitating access to services and coordinating care, yet their role in neuro-oncology remains poorly characterized. This scoping review examines the structure, delivery, and reported outcomes of patient navigation programs for individuals with CNS tumors.

**Methods:**

We conducted a comprehensive literature search of Medline, Embase, PsycINFO, and Cochrane Central to identify studies published from 1990 onward describing patient navigation interventions for adults with primary CNS tumors or brain metastases. Data were extracted on study design, intervention focus, navigator role, delivery modality, population characteristics, and reported outcomes.

**Recent Findings:**

Twenty-two studies met inclusion criteria, including 15 conference abstracts and 7 peer-reviewed journal articles. Navigation programs varied in structure but commonly addressed care coordination, patient education, emotional support, financial navigation, and access to clinical trials. Services were delivered in person, by telephone, or through hybrid models. Navigators included nurses, social workers, clinical trial coordinators, and non-clinical staff. Reported outcomes included improved care transitions, increased patient satisfaction, enhanced emotional well-being, reduced emergency department utilization, and, in some programs, reduced financial burden and increased clinical trial enrollment. Barriers included cognitive limitations, challenges in outcome measurement, and inconsistent documentation.

**Summary:**

Patient navigation programs in neuro-oncology show promise in addressing complex patient needs, but additional high-quality outcomes data are needed to inform future implementation.

## Introduction

Originally developed to address inequities in access to care, patient navigation helps patients across diverse populations overcome barriers to treatment, improve quality of life, and increase satisfaction with care [1]. Dr. Harold Freeman introduced the concept in 1990, launching a program that targeted obstacles to timely cancer screening and treatment [2, 3]. The program improved outcomes, leading to earlier cancer detection, diagnosis at earlier stages, and faster initiation of treatment [3]. This work inspired the *Patient Navigator and Chronic Disease Prevention Act* of 2005, which established patient navigation as a formal intervention [4]. Over the past 30 years, patient navigation has evolved into a key strategy to reduce health care inequities by providing patient-centered support, and it now represents an essential component of comprehensive care [5]. Navigation programs enhance the timeliness of care, participation in cancer screening, and diagnostic follow-up, particularly among systemically marginalized groups such as those with limited English proficiency [6–8]. Reflecting its growing role, the Centers for Medicare & Medicaid Services (CMS) designated patient navigation as a reimbursable service beginning in 2024 [9].

The evidence supporting patient navigation is strongest in breast, cervical, colorectal, and prostate cancers. The NCI’s Patient Navigation Research Program (PNRP) was one of the first large efforts to evaluate navigation using a consistent approach across multiple sites. The program established a common definition of patient navigation and focused on outcomes such as time to diagnostic resolution, time to treatment, patient satisfaction, and cost-effectiveness [10]. These measures largely focused on the period between an abnormal screening result and the start of treatment. In a later study of more than 10,000 participants, many of whom were from racial and ethnic minority groups and were publicly insured or uninsured, navigation was associated with improvements in diagnostic resolution (aHR = 1.51) and treatment initiation (aHR = 1.43) after 90 days. The benefit was greater at sites where patients experienced longer delays in usual care [11]. While the PNRP helped establish the evidence for patient navigation, its outcomes focused mainly on diagnosis and treatment initiation. Navigation programs now often continue into survivorship and palliative care, which may require a broader set of measures to evaluate their impact.

Although patient navigation is now common in oncology, few studies have examined its impact among patients with central nervous system (CNS) tumors. CNS tumors include both primary and secondary neoplasms of the brain and spinal cord. Primary CNS tumors account for less than 2% of all cancers diagnosed annually in the United States [12]. Yet, they contribute disproportionately to cancer-related morbidity and mortality, often carrying a poor prognosis [12, 13]. Secondary CNS tumors, most commonly brain metastases, are far more prevalent and also contribute substantially to both symptom burden and cancer mortality [14]. CNS tumors can cause cognitive impairment, aphasia, motor deficits, visual deficits, seizures, and neuropsychiatric symptoms, among others [13]. They may also limit opportunities for clinical trial enrollment, as such deficits can be perceived as reducing patients’ ability to provide informed consent or meet eligibility criteria [15, 16]. Patients with CNS tumors require coordinated and multidisciplinary support to manage these complex needs [17].

Social determinants of health (SDOH), the non-medical conditions that shape health outcomes, often compound these neurologic challenges [18, 19]. Patients from systemically marginalized populations face additional barriers that contribute to persistent health disparities [20]. For individuals with CNS tumors who also experience structural disadvantage, these overlapping challenges underscore the need for interventions that can bridge gaps in care [21–24]. Given this context, this scoping review maps existing patient navigation programs and interventions for individuals with CNS tumors, and identifies their core elements, delivery models, and reported outcomes to guide future research and the development of tailored, multidisciplinary navigation strategies.

### Review question

What are the reported characteristics, barriers, facilitators, and outcomes of patient navigation programs designed to support people with central nervous system tumors?

### Methods

We conducted a scoping review to comprehensively map and synthesize all relevant available literature related to patient navigation programs or interventions for patients with CNS tumors. This approach allowed us to assess existing research, identify knowledge gaps, establish trends, and summarize existing programs to inform future program development. The review followed the Preferred Reporting Items for Systematic reviews and Meta-Analyses extension for Scoping Reviews (PRISMA-ScR) guidelines to ensure reporting transparency [25].

### Search strategy

A medical librarian designed a search strategy using keywords and subject headers on three main topics: patient navigation, intervention focus, and central nervous system/brain cancers. After implementation in Medline ALL (Ovid), a second librarian performed a PRESS review [26]. The strategy was then translated to Embase (Elsevier), PsycInfo (Ovid), and Cochrane Central (Wiley) with the aid of the Systematic Review Accelerator’s Polyglot tool [16]. Results were limited to items in English published from 1990 onward. Searches were run on September 26, 2024, and results were uploaded to the systematic review tool Covidence (Veritas Health Innovation), where duplicates were automatically removed. Detailed search strategies for all databases are provided in Appendix 1 and 2.

### Eligibility criteria

We defined patient navigation for individuals with CNS tumors as any program or intervention delivered by a trained navigator, healthcare professional, or technology-based platform that supports patients in overcoming barriers to care, coordinating services, and accessing diagnostic, therapeutic, or supportive resources. Eligible studies included programs for adults (≥ 18 years) with a current or prior diagnosis of a CNS tumor, including primary and secondary cancers. Studies focused solely on anti-tumor treatments, including medical, surgical, or radiation therapies, were excluded. Outcomes of interest included hospital readmissions, emergency department visits, appointment adherence, completion of treatment, medication compliance, needs addressed, care plan development, quality of life, and feasibility. We included studies published in English and included randomized controlled, cohort, qualitative, mixed-methods, longitudinal, or quality improvement designs, as well as meeting abstracts. Editorials, unpublished data, and reviews or meta-analyses without empirical findings were excluded. Eligible programs could be hospital-based or community-based.

### Data analysis and presentation

We extracted the following information: author, date of publication, intervention type, intervention focus, study design, target population characteristics (age, diagnosis, socioeconomic status, health insurance coverage), and reported limitations of the intervention or study design. Intervention type referred to the mode of delivery, while intervention focus described the targeted health outcome, disparity, resource utilization, or patient need.

We recorded team composition and navigator titles to determine whether programs met our definition of patient navigation. We also reviewed the program delivery format, outcomes assessed, and patient experience to confirm eligibility for navigation interventions and to evaluate the reported results. Finally, we extracted patient-reported needs and experiences to assess program effectiveness and overall patient response.

## Results

### Study inclusion

**Fig. 1.**
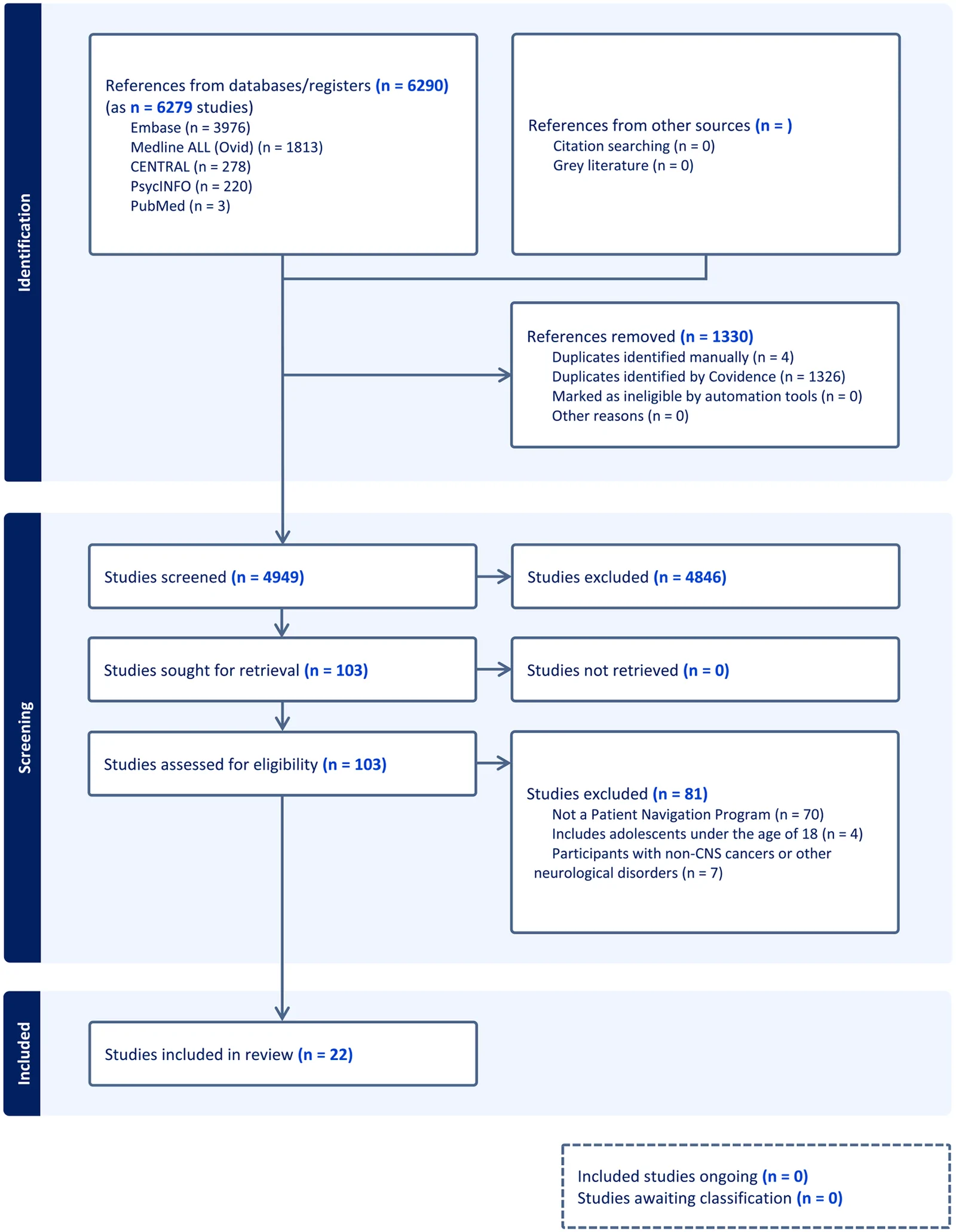
PRISMA-ScR flow diagram illustrating the search results, screening, and inclusion process.

A total of 6290 references, representing 6,279 studies, were retrieved through the search strategy. These records were uploaded to Covidence, where 1330 duplicates were removed, leaving 4949 abstracts for screening. Of these, 4846 were excluded, and 103 full texts were retrieved for eligibility assessment. Most of the excluded full-text studies did not include a patient navigation component, while others focused on non-CNS cancer populations. In total, 22 studies met the inclusion criteria and were included in this scoping review. Fig. 1 summarizes the search strategy, source selection, and inclusion process.

### Characteristics of included sources

**Fig. 2.**
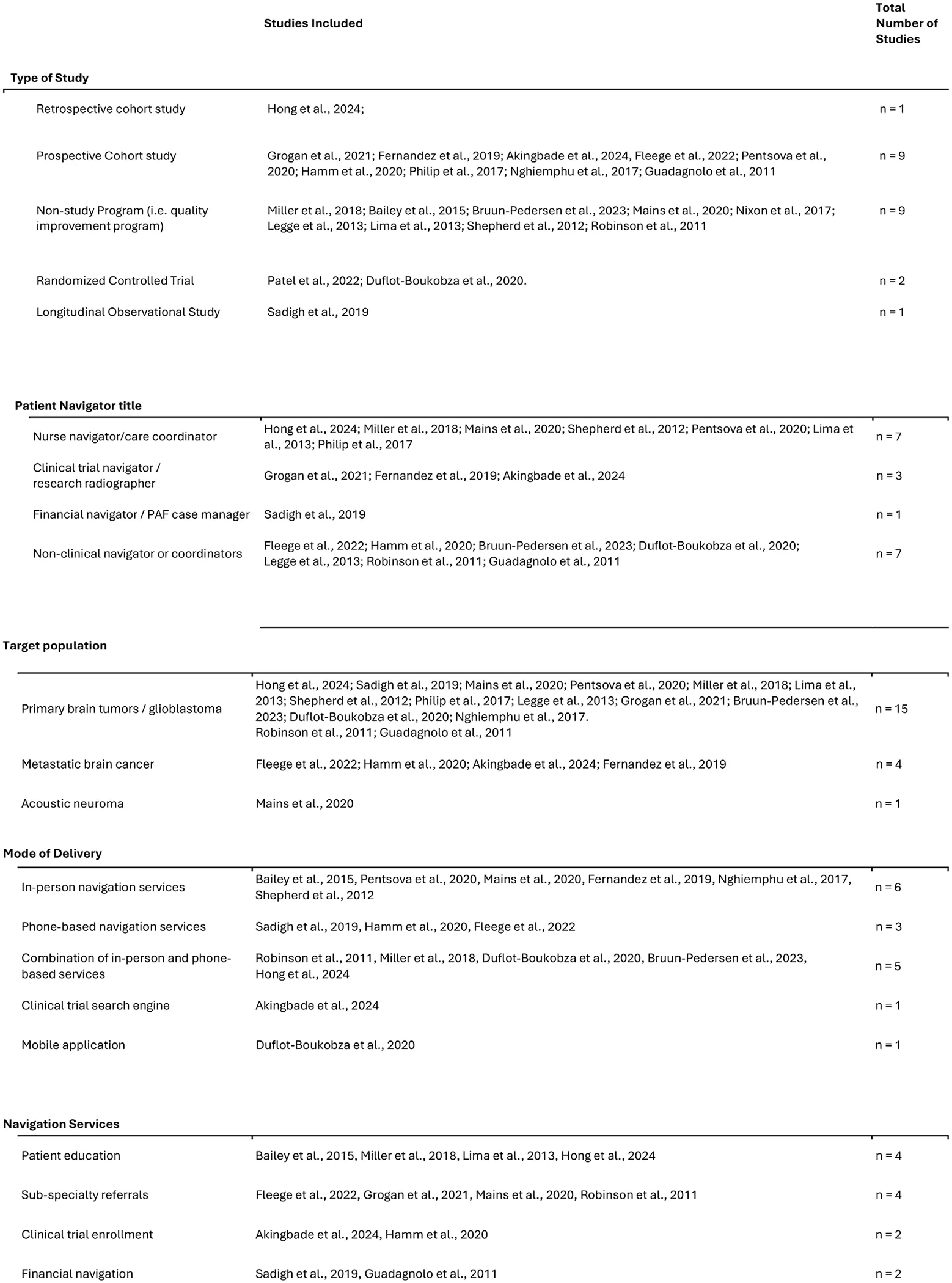

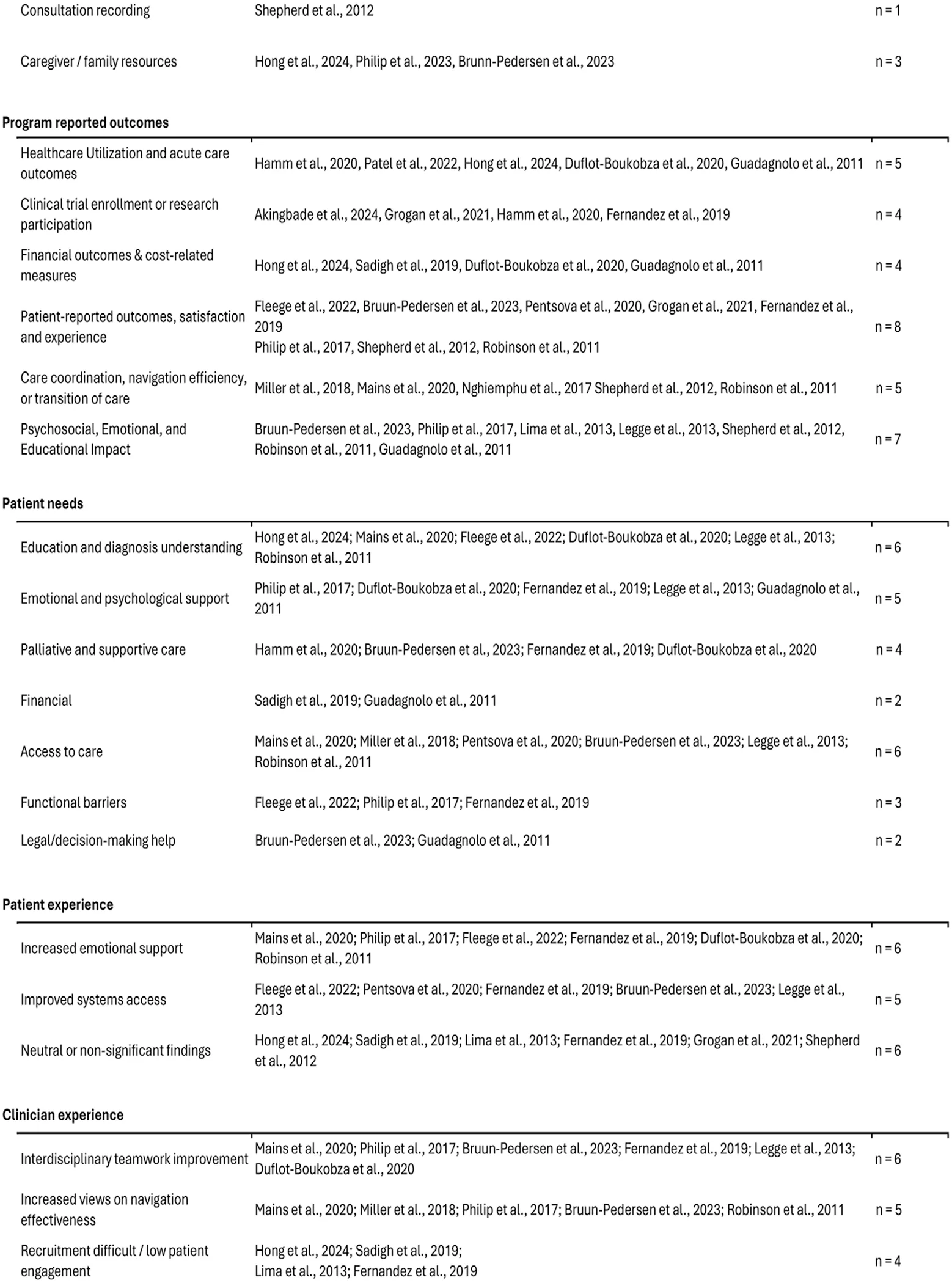
Table of trends in patient navigation program structure, delivery and outcome.

Although the search encompassed studies published from 1990 onward, all studies meeting the inclusion criteria were published between 2011 and 2024. The included sources comprised prospective cohort studies (n = 9), non-study programs (i.e. quality improvement interventions) (n = 9), randomized controlled trials (n = 2), a retrospective cohort study (n = 1), and a longitudinal observational study (n = 1). A majority of these were conference abstracts (n = 15), with the remainder published as full journal articles (n = 7). Across studies, sample sizes were generally modest, a priori power or sample size justification was infrequently reported, and clinical and patient-centered outcomes were variably captured, which may limit interpretability. An overview of program characteristics and key components is shown in Fig. 2.

### Program descriptions and services

All twenty-two studies described a single patient navigation program. Programs were based in the United States (n = 10), Australia (n = 5), Canada (n = 2), United Kingdom (n = 2), France (n = 1), and Denmark (n = 1). Together, these studies represented programs serving urban, suburban, and rural populations.

All reviewed programs provided care coordination and linkage to health or supportive services, typically initiated by a referral from a provider or social worker. Most offered guidance to patients and caregivers on accessing resources, including clinical trials, psychosocial assessments, and financial assistance. Enrollment generally began with an intake or baseline survey collecting demographic data, followed by a needs assessment addressing financial concerns, symptom burden, treatment adherence, psychosocial support, and education needs. In financial navigation programs, surveys assessed financial toxicity, defined as the objective financial burden and subjective psychosocial stress from care costs [27–29].

Navigation services included connecting patients to appropriate services, facilitating provider communication, managing symptoms, and scheduling or triaging appointments. Eight programs featured nurse or clinical navigators who coordinated care for brain tumor patients through phone calls, in-person visits, or structured planning tools [30–37]. Programs commonly provided educational support through handouts, digital tools, or one-on-one coaching. Five programs incorporated patient-reported outcome tracking and ongoing needs assessments, allowing teams to adapt services to changing patient needs [38–42]. Two programs incorporated financial navigation, linking patients with case managers for insurance, costs, and financial aid support [37, 39]. Others supported research participation or clinical trial enrollment (n = 3) [42–44], while several offered psychosocial support or emotional support, particularly for young adults or high-grade gliomas. Studies also emphasized timely referrals and end-of-life coordination [31, 34, 38, 40, 42, 44].

### Patient navigator title

Among studies that reported team composition, most included an interdisciplinary team of oncologists, neurologists, psychiatrists, physical therapists, social workers, case managers, and patient navigators. Navigator background varied; some had clinical training, others were non-clinical staff managing logistics and referrals. Eight studies involved nurse navigators, with titles such as ‘nurse navigator’, ‘nurse care coordinator’, or ‘Brain Cancer Care Coordinator’ [30–33, 35, 45, 46]. Three studies focused on research participation, using ‘clinical trial navigators’ or ‘neuro-oncology research radiographers.’[43, 44,47] One financial navigation study used a Patient Advocate Foundation (PAF) case manager [39]. The remaining seven studies used non-specific navigator titles for non-clinical staff supporting referrals and communication [37,38,42,45,48–50].

There was no apparent relationship between navigator title and program setting, as both ‘coordinator’ and ‘navigator’ titles were used in community neighborhood programs and clinical settings. However, there was a relationship between program focus and navigator background: programs addressing clinical trial enrollment, financial support (managing living expenses such as rent, bills, groceries), and symptom management generally employed navigators with experience in research, personal finance, and clinical practice, respectively.

### Patient population

Based on the inclusion criteria, all studies included patients with a primary brain or spinal cord tumor or with brain or spine metastases (Appendix 3). Most studies (n = 15) focused specifically on brain tumors, most commonly gliomas [30–37,39,44–46,48,49,51]. Program eligibility criteria often included adults (≥ 18) with a brain cancer diagnosis who were newly diagnosed or actively undergoing treatment with surgery, radiation, or chemotherapy. Several studies (n = 4) included individuals with brain metastases from systemic cancers [38,40,43,50], and three studies were pan-cancer studies that allowed enrollment of patients with CNS tumors [43,50,52]. One study focused on acoustic neuroma [35] and another specifically targeted an adolescent and young adult (AYA) population (ages 18–35) [33]. Three studies also required referral to a navigation program, enrollment within defined timeframes, or participation in a care model or clinical trial [43,48,52].

### Study population size

Across the included studies, sample sizes ranged from small pilot cohorts to large, multi-cancer navigation programs; participant counts for each individual study are reported in Appendix 3. Brain tumor–specific studies were generally modest in size (n = 12–202). Most enrolled fewer than 60 participants, including Sadigh et al., Fernandez et al., Bruun-Pedersen et al., Shepherd et al., Philip et al., Grogan et al., and Duflot-Boukobza et al.; a few mid-sized studies enrolled 60 to 130 participants (Fleege et al., Pentsova et al., and Robinson et al.); and only two CNS-only studies exceeded 150 participants, Hong et al. (n = 187) and Nghiemphu et al. (n = 202). Several non-study programs did not report participant numbers. In contrast, larger multi-cancer studies (n = 96–373), including Akingbade et al., Patel et al., Hamm et al., and Guadagnolo et al., included brain cancer only as a subset of a broader oncology population, limiting the ability to draw CNS-specific conclusions from their overall sample sizes.

### Mode of delivery

The patient navigation programs included in this review were delivered through in-person, telephone-based, web-based, or hybrid approaches (Appendix 4). Six programs were conducted primarily in-person, integrating navigation into outpatient or postoperative visits with scheduled follow-ups to coordinate ongoing care [32,35,37,44,45,48]. Three programs were delivered primarily by telephone, providing intake screening, follow-ups, referrals, financial management, and clinical trial discussions [38,39,52]. Five programs used hybrid models that combined in-person, telephone, telehealth, or video call interactions [30,31,33,34,46]. A common feature of hybrid programs was the use of phone-based and/or web-based platforms for enrollment, screening, and communication, with in-person visits reserved for patient education, symptom management, and emotional support. One study, designed to improve clinical trial access, reviewed patients’ medical records to assess eligibility and searched multiple trial databases to identify suitable matches [43]. Another used a mobile application to facilitate communication between nurse navigators and patients, focusing on symptom management and toxicity monitoring [34].

### Navigation services

The services offered by patient navigation programs varied depending on program goals, such as reducing emergency department visits and hospitalizations, improving quality of life, facilitating financial stability, and aiding in access to clinical trials. Patient education was a key service, delivered through printed materials, in-person sessions, and web-based platforms, where providers or nurse navigators educated patients and caregivers about diagnosis, treatment, and wellness resources [30–32,49]. Three programs explicitly incorporated caregiver support into their navigation services. Hong et al. provided comprehensive support to patients and their families throughout diagnosis, treatment, clinical trials, symptom management, community follow-up, and end-of-life care through Brain Cancer Care Coordinators [28]. Philip et al. incorporated family caregiver engagement at key transition points, including post-diagnosis, post-diagnostic hospitalization, and post-radiotherapy, while Bruun-Pedersen et al. provided ongoing support for young adults with brain tumors and their caregivers through regular follow-up with a nurse care coordinator [31,45]. One program helped patients create question lists and provided audio recordings of their consultations with their oncologist to ensure ongoing access to information [37]. Specialist referral was another common service, with four programs reporting referrals to specialty services, including rehabilitation medicine and palliative care 35, 38, 40, 46 Several programs focused on clinical trial access, often using non-medical navigators and physicians to match patients to clinical trials using chart review and trial database searches [33,48,47]. Financial navigation was also emphasized, as some programs incorporated financial toxicity screening followed by navigator assistance with financial aid, travel coordination, and insurance communication [39,50].

### Patient needs and barriers to care

Across the studies, patients and caregivers reported a wide range of needs. A common theme was the need for clear information and edcation, including support in understanding diagnoses, treatment options, and care planning [30,32,37,48,46,49]. Psychosocial and emotional needs were also frequently reported, such as anxiety, reduced hope, and the need for emotional reassurance, particularly among young adults and caregivers [33,37,47,50,53]. Financial strain and insurance issues were described in two studies, including debt, treatment-related cost burdens, and difficulties navigating insurance systems [39,50]. Six studies addressed barriers to care coordination and system access, especially in arranging appointments, transitioning between services, or accessing specialty care [31,32,35,37,45,46]. Supportive and palliative care needs included assistance with goals of care, referrals to palliative services, and end-of-life planning [40,42,48,47]. Functional and rehabilitation care needs were noted, particularly related to physical decline, fatigue, and barriers to therapy [33,38,42]. Two studies documented legal and decision-making needs, including support with advance directives and health proxies [48,50].

### Program reported outcomes

The reported outcomes across navigation programs spanned clinical, psychosocial, and system-level goals (Appendix 5). Five studies evaluated healthcare utilization, including emergency department visits, hospitalizations, and cost savings [30,34,40,50]. Four studies focused on clinical trial enrollment or research participation, measuring referral success, patient eligibility, and recruitment efficiency [42–44,52]. Four studies assessed financial outcomes using pre- and post-program surveys of financial toxicity [30,34,39,50]. Eight programs assessed patient-reported outcomes (PROs) such as quality of life, caregiver burden, and emotional well-being [33,37,38,42,44,48,46,47]. Several studies examined care coordination and care transitions, particularly in postoperative recovery and timely follow-up [31,35,37,45,46]. Seven studies measured psychosocial and educational impacts, including reduced anxiety, improved coping, and greater preparedness for care [33,37,46,47,49–51].

#### Survival and Clinical Outcomes:

Many studies did not report survival or clinical outcome data. Among those that did, Hong et al., which provided longitudinal support across diagnosis, treatment, and end-of-life care, reported no significant difference in overall survival among glioblastoma patients; however, the use of brain cancer care coordinators was associated with fewer emergency visits, fewer hospital admissions, and a twenty-four percent reduction in hospital length of stay 28. Akingbade et al. evaluated a program that referred patients for clinical trial enrollment and reported an increase in median survival from 3.0 to 5.3 months among referred patients, though this finding may have been confounded by trial participation patterns 41. Additionally, Duflot-Boukobza et al. found that their intervention decreased emergency department visits and hospitalizations, and increased supportive care utilization [32].

#### Access and Care Coordination:

Miller et al. reported an increase in the proportion of patients with glioblastoma initiating radiotherapy within 35 days of surgery, from 43% to 60%, compared with historical institutional data 29. Grogan et al. observed that 40% (17 of 43) of patients pursued clinical research participation, and 66% saw subspecialists, resulting in 56 new specialty referrals through a care coordination program for metastatic breast cancer with CNS involvement [42]. Hamm et al. found that 69% of patients completed follow-up with a clinical trials navigator, although 25% died before referral [52]. Nghiemphu et al. and Guadagnolo et al. also reported improved coordination and timeliness of patient follow-up [45,50].

#### Financial and Psychosocial Support:

Some programs addressed both financial and psychosocial challenges. Sadigh et al. conducted a small study of 12 patients, reporting reduced financial hardship and $15,110 in debt relief through navigator-led case management [39]. Bailey et al. and Bruun-Pedersen et al. reported reduced anxiety, greater emotional comfort, and stronger support for patients and caregivers [32,33]. Philip et al. observed improvements in quality of life and reduced unmet informational needs among high-grade glioma patients and their caregivers [47]. Legge et al. found higher levels of hope among patients not receiving active treatment, although greater symptom burden correlated with lower hope [51]. Nixon et al. reported reduced distress and improved treatment adherence, while Shepherd et al. observed better patient understanding during consultations [36,46].

### Patient experience

Patient experiences across the navigation programs were generally positive, though some studies reported mixed or neutral outcomes. Many patients described greater emotional support and coping capacity, with reduced anxiety and improved comfort in care settings, particularly among young adults, caregivers, and those receiving coordinated outpatient services [32,33,37,48,47,53]. Five studies reported improved access to care and system navigation, including quicker referrals, smoother transitions between providers, and enhanced preparation for oncology consultations [31,38,42,45,46]. High satisfaction was also frequently reported, with patients and caregivers noting consistency, clarity, and usefulness of their interactions with navigators [37,44,48,47]. Nghiemphu et al., Miller et al., and Pentsova et al. (2020) all reported high patient and provider satisfaction, with Nghiemphu et al. noting increased Press Ganey scores following implementation of a navigation program [31,48,45]. Some studies found neutral or non-significant results, such as no statistical difference in patient costs or satisfaction, often attributed to small sample sizes, incomplete follow-up, cognitive decline, or high mortality [30,36,39,40,42,52]. Specific tools, such as patient education notebooks, consultation audio recordings, and personalized calendars, were generally perceived as helpful for tracking symptoms and clarifying care decisions [44,46,49].

### Clinician and navigator experience

Clinicians and navigators reported both benefits and challenges of program implementation. Many studies described improved interdisciplinary communication, workflow, and coordination across specialties when navigators were part of multidisciplinary teams [31,33,44,45,48,49]. Several studies noted positive perception of program effectiveness, highlighting the role of navigators in bridging gaps in care, increasing access to services, and enhancing the overall quality of support [31–33,37,48]. Clinicians in four studies also identified opportunities for improvement, including insufficient documentation of navigator interactions [30], recruitment challenges [39,40], and low patient engagement with outcome measures [42]. Additional barriers included limited clinic space, high patient mortality, caregiver dependence, and increased needs for financial or psychosocial assistance [39,40,49,50]. Clinician or team experience was not reported in seven studies, either because focus was exclusively on patient or caregiver outcomes or because no evaluative feedback from the care team was included [34–36,38,46,47,53].

## Discussion

This scoping review highlights that patient navigation in neuro-oncology remains nascent, with few published manuscripts and most data limited to conference abstracts. By comparison, a recent systematic review of patient navigation in oncology included 59 full-text articles and was able to exclude conference abstracts [54]. The limited evidence base underscores a major research gap in how navigation can best support patients with CNS tumors, a population with unique clinical and psychosocial needs [16,18,20].

Programs in this review used broad and heterogeneous measures of success, spanning health care utilization, psychosocial outcomes, and financial toxicity. While this breadth reflects the adaptability of navigation, it limits comparability across studies. Many programs were quality-improvement initiatives, rather than randomized or controlled evaluations, making it difficult to compare or generalize outcomes. Future research should prioritize methodologically rigorous, adequately powered studies that can identify where and for whom navigation provides the greatest benefit [55]. An additional limitation of the current evidence is the inconsistent reporting of patient demographic and socioeconomic characteristics. Although patient navigation was originally developed to reduce disparities in access to care, many studies did not report participant characteristics such as race and ethnicity, insurance status, socioeconomic factors, or other social determinants of health. This limited our ability to evaluate whether existing neuro-oncology navigation programs are effectively reaching populations most affected by disparities in care. Future studies should routinely report these characteristics to better understand equity in program implementation and outcomes.

Hybrid and telehealth-based navigation models emerged after 2015, reflecting broader shifts in oncology toward virtual care [56,57]. These models may be particularly well-suited to patients with neurologic disability or those living far from specialty centers. As digital health and artificial intelligence evolve, new tools could extend the reach of navigation and enable proactive symptom monitoring [58]. Other oncology fields, such as breast oncology, have consistently integrated mobile applications and digital tools into navigation, providing a model for CNS programs to build upon [59,60]. However, these technologies may also introduce new barriers related to digital literacy, access to technology, language, disability, and data privacy, potentially widening disparities among patients already at greatest risk for unmet needs [56]. As AI-enabled navigation develops, implementation should therefore incorporate equity-focused safeguards, including representative data, transparency and accountability, patient and community engagement, and ongoing assessment for differential performance across populations.

Funding and sustainability of patient navigation programs remain critical challenges, particularly amid rising costs of neuro-oncologic care and dependence on philanthropic support [61]. Some clinical trials reported dedicated funding, and a few quality-improvement initiatives described programmatic backing, but most did not specify long-term or sustainable financial support. As patient navigation becomes increasingly adopted and eligible for reimbursement, future work should evaluate whether CNS programs are leveraging these mechanisms to promote sustainability and integration into standard care [62].

Economic evaluations of patient navigation outside of neuro-oncology offer a useful benchmark for future cost-effectiveness research in this population. In the PNRP, a formal cost-consequence analysis found that navigation added approximately $275 per patient and produced only modest gains in the probability of diagnostic resolution, concluding that navigation was likely to be cost-effective only if improved resolution translated into an earlier stage at diagnosis [63]. In contrast, a modeling study of patient navigation for screening colonoscopy among minority populations found that one-time navigation could be cost-saving, and that navigation embedded in a longitudinal screening program cost approximately $9800 per quality-adjusted life-year gained, well within accepted cost-effectiveness thresholds [64]. These findings suggest that the economic value of navigation depends heavily on clinical context, population risk, and the downstream outcomes it influences. No comparable cost-effectiveness analysis has yet been conducted for neuro-oncology navigation programs; given the funding and sustainability challenges described above, economic evaluation should be a priority for future CNS-focused navigation research.

This scoping review mapped the current landscape of patient navigation programs for CNS tumors. A key strength was the inclusion of both research studies (including randomized clinical trials and cohort studies) and non–study programs (such as quality improvement initiatives), enabling a more comprehensive characterization of real-world program implementation. By examining program context and structure, this review provides insight into how navigation is currently delivered in neuro-oncology. However, variability in study quality and detail, particularly among abstracts, limited the consistency of available data. Moving forward, more rigorous research is needed to evaluate effectiveness and guide implementation tailored to the unique challenges of CNS cancer. Standardizing outcome measures and defining the core components of effective navigation will be essential to advancing equitable, patient-centered neuro-oncologic care.

## Conclusions

This scoping review suggests that patient navigation programs show promise for patients with CNS tumors and can help address social determinants of health in this population. Reported outcomes were generally positive, with patients further finding navigation beneficial in terms of support, coordination, and access to resources. Future research should evaluate not only patient-reported and clinical outcomes, but also the feasibility and long-term sustainability of implementation. Although relatively new in neuro-oncology, patient navigation likely represents a promising strategy to enhance care and support for patients with CNS tumors.

## Key Take Home Points:

Evidence on patient navigation in CNS tumors is limited but suggests potential benefits in improving care coordination, access to services, and patient experience.

Navigation programs address multidimensional needs, including coordination of complex care, psychosocial support, and financial and logistical challenges faced by patients and caregivers.

Standardized outcomes and more rigorous studies are needed to establish effectiveness and define best practices to guide broader implementation.

## Importance of the Study

Patients with central nervous system tumors face unique clinical, cognitive, and psychosocial challenges that complicate cancer care delivery. Although patient navigation is well established in oncology, its application in neuro-oncology has not been systematically characterized. This scoping review provides the first comprehensive synthesis of patient navigation programs designed for individuals with CNS tumors, including both primary brain tumors and brain metastases. By mapping program structures, navigator roles, delivery models, and reported outcomes, this study highlights the breadth of existing approaches and identifies key gaps in evidence, including the limited availability of high-quality outcomes data and inconsistent measurement methods. The findings underscore the promise of navigation in addressing social determinants of health, improving care coordination, and supporting patients and caregivers facing neurologic disability. This work lays a foundation for future research to develop standardized, sustainable, and rigorously evaluated navigation interventions tailored to the complex needs of neuro-oncology populations.

## Appendix I. Search strategy.

Medline ALL (Ovid) Search, 9/26/24

1946 to September 25, 2024

1813 Results

**Table:**

1	case managers/ or comprehensive health care/ or exp “continuity of patient care”/ or hospital to home transition/ or house calls/ or patient care team/ or patient navigation/ or transitional care/	378,270
2	(adherence support* or aftercare* or after-care* or assistance program* or coordinated approach* or navigator* or social prescri* or support* discharg*).ti,ab,kf.	21,287
3	((case or care) adj4 (comanage* or co-manage* or embed* or integrat* or management or manager* or map* or model or models or network* or pattern* or partnership* or transition*)).ti,ab,kf.	189,668
4	((admiral or coordinat* or co-ordinat* or navigat* or phased or pivot) adj1 nurs*).ti,ab,kf.	1441
5	((home* or house*) adj2 (call* or visit*)).ti,ab,kf.	16,375
6	((advoca* or centrali* or collaborat* or coordinat* or co-ordinat* or continuit* or cooperat* or navigat* or pathway* or path-way* or shared) adj4 (care or consult* or health* or intervention or interventions or partnership* or program* or service*)).ti,ab,kf.	139,435
7	(comprehensive* adj4 (care or consult* or followup* or follow* up or follow ups or intervention or interventions or management or network* or partnership* or program* or service*)).ti,ab,kf.	53,784
8	((caregiv* or care giver* or care giving or carer* or communit* or dyad* or families or family or outpatient* or patient* or personal) adj2 (continuit* or coordinat* or co-ordinat* or mapping or navigat* or pathway* or path-way* or team* or transition*)).ti,ab,kf.	36,643
9	((biopsychosocial or bio-psychosocial or bio-psycho-social or crossdepartment* or cross-department* or crossdisciplin* or cross-disciplin* or crossprofession* or cross-profession* or interdepartment* or inter-department* or interdisciplin* or inter-disciplin* or interprofession* or inter-profession* or multidepartment* or multi-department* or multidisciplin* or multi-disciplin* or multimodal or multi-modal or multiprofession* or multi-profession*) adj2 (approach* or care or case* or collaboration* or coordinat* or co-ordinat* or continuit* or continuum* or cooperat* or facilitat* or guide or guides or healthcare or integrat* or intervention* or link* or navigat* or network* or neurorehab* or neuro-rehab* or partnership* or pathway* or path-way* or program* or rehab* or service* or support* or team* or transition*)).ti,ab,kf.	136,004
10	((app or apps or digital or discharg* or ehealth or e-health or internet or mhealth or m-health or (mobile adj3 health*) or online or remote* or phone* or smartphone* or telephone*) adj3 (followup* or follow* up or follow ups or intervention or interventions)).ti,ab,kf.	37,217
11	1 or 2 or 3 or 4 or 5 or 6 or 7 or 8 or 9 or 10	866,718
12	exp “Activities of Daily Living”/ or exp Community Health Services/ or Community Integration/ or exp Insurance/ or exp “Facilities and Services Utilization”/ or exp Health Inequities/ or exp Health Services Accessibility/ or Home Care Services/ or “Home Care Services, Hospital-Based”/ or Home Health Nursing/ or Home Nursing/ or Homemaker Services/ or Medically Uninsured/ or Needs Assessment/ or exp “Patient Acceptance of Health Care”/ or exp Patient Care Planning/ or Patient-Centered Care/ or Patient Readmission/ or exp “Public Assistance”/ or “Public-Private Sector Partnerships”/ or exp “Quality of Life”/ or “Return to School”/ or “Return to Work”/ or Self-Management/ or exp Social Support/ or exp “Treatment Adherence and Compliance”/	1419,710
13	((reenter* or re-enter* or reentr* or re-entr* or return*) adj2 (college* or job or jobs or school* or universit* or work*)).ti,ab,kf.	17,304
14	(advance* directive* or advance* plan* or care goal* or care plan* or daily life or daily living or financ* or food* or functional status* or “goals of care” or housing or HRQOL or insur* or living situation* or malnourish* or mal-nourish* or malnutrition or mal-nutrition or Medicaid or Medicare* or monetar* or need* assessment* or psychosocial* or psycho-social* or QOL or (quality adj3 life) or readmission* or re-admission* or readmit* or re-admit* or reemploy* or re-employ* or rehospital* or re-hospital* or reintegrat* or re-integrat* or self care or self manag* or SNAP or supplemental nutrition or underinsur* or unemploy* or unempower* or uninsur* or WIC).ti,ab,kf.	1868,811
15	((caregiv* or care giver* or care giving or carer* or communit* or dyad* or families or family or outpatient* or participant* or patient* or person or persons or personali* or population* or proxies or proxy or public) adj3 (access* or adher* or aid or aide* or assist* or barrier* or burden* or centered* or centred* or comply or complian* or connect* or employment or educat* or empower* or enroll* or experience or experiences or integrat* or job or jobs or nonadher* or noncomplian* or partner* or preference* or recruit* or support* or workplace* or work place* or workforce* or work force*)).ti,ab,kf.	940,113
16	12 or 13 or 14 or 15	3404,055
17	exp central nervous system neoplasms/ or exp neoplasms, neuroepithelial/	287,549
18	exp central nervous system/ and exp neoplasms/	84,739
19	(astroblastoma* or astrocytoma* or ATRT* or atypical teratoid rhabdoid tumo* or chondroma* or chondrosarcoma* or ependymoma* or esthesioneuroblastoma* or gangliocytoma* or ganglioglioma* or ganglioneuroblastoma* or glioma* or glioblastoma* or glio-blastoma* or gliosarcoma* or glio-sarcoma* or haemangiopericytoma* or haemangio-pericytoma* or hemangiopericytoma* or hemangio-pericytoma* or liponeurocytoma* or medulloblastoma* or medullo-blastoma* or medulloepithelioma* or medullo-epithelioma* or meningioma* or neuroblastoma* or neuro-blastoma* or neurocytoma* or neuro-cytoma* or neuroepithelioma* or neuro-epithelioma* or neuroleukemi* or neuro-leukemi* or neuroleukaemi* or neuro-leukaemi* or neurooncolog* or neuro-oncolog* or oligoastrocytoma* or oligodendrocytoma* or oligo-dendrocytoma* or oligodendro-cytoma* or oligo-dendro-cytoma* or oligodendroglioma* or oligo-dendroglioma* or PCNSL* or pineoblastoma* or pineoblastoma* or pineocytoma* or pineocytoma* or rhabdomyosarcoma* or rhabdo-myosarcoma* or rhabdomyo-sarcoma* or subependymoma* or teratoid or xanthoastrocytoma*).ti,ab,kf.	234,074
20	((brain* or CNS or central nervous system* or cerebr* or cranial* or cranium* or dura or dural or extradura* or glia* or intracrani* or intra-crani* or intradura* or intramedul* or intra-medul* or leptomenin* or lepto-menin* or mater or mening* or meninx* or optic nerve* or paramenin* or paranasal* or para-nasal* or pineal* or pituitar* or spine or spines or spinal) adj3 (blastoma* or cancer* or carcinoma* or leukemi* or leukaemi* or lymphoma* or malignan* or metasta* or neoplasm* or oncolog* or sarcoma* or tumor or tumour or tumors or tumours)).ti,ab,kf.	174,324
21	17 or 18 or 19 or 20	452,076
22	11 and 16 and 21	1981
23	limit 22 to (english language and yr=“1990 -Current”)	1813

Embase (Elsevier) Search, 9/26/24

3973 Results

**Table:**

1	‘aftercare’/de OR ’care coordinator’/de OR ‘case management’/de OR ‘case manager’/de OR ‘collaborative care team’/de OR ‘hospital to home transition’/de OR ‘home visit’/de OR ‘integrated health care system’/de OR ‘multidisciplinary team’/de OR ‘patient care team’/de OR ‘social prescribing’/de OR ‘transitional care’/de	89,827
2	‘adherence support*’:ti,ab,kw OR aftercare*:ti,ab,kw OR ‘after care*’:ti,ab,kw OR ‘assistance program*’:ti,ab,kw OR ‘coordinated approach*’:ti,ab,kw OR navigator*:ti,ab,kw OR ‘social prescri*’:ti,ab,kw OR ‘support* discharg*’:ti,ab,kw	31,193
3	((case OR care) NEAR/4 (comanage* OR ‘co manage*’ OR embed* OR integrat* OR management OR manager* OR map* OR model OR models OR network* OR pattern* OR partnership* OR transition*)):ti,ab,kw	267,895
4	((admiral OR coordinat* OR ‘co ordinat*’ OR navigat* OR phased OR pivot) NEAR/1 nurs*):ti,ab,kw	3009
5	((home* OR house*) NEAR/2 (call* OR visit*)):ti,ab,kw	21,883
6	((advoca* OR centrali* OR collaborat* OR coordinat* OR ‘co ordinat*’ OR continuit* OR cooperat* OR navigat* OR pathway* OR ‘path way*’ OR shared) NEAR/4 (care OR consult* OR health* OR intervention OR interventions OR partnership* OR program* OR service*)):ti,ab,kw	195,150
7	(comprehensive* NEAR/4 (care OR consult* OR followup* OR ‘follow* up’ OR ‘follow ups’ OR intervention OR interventions OR management OR network* OR partnership* OR program* OR service*)):ti,ab,kw	77,610Cl
8	((caregiv* OR ‘care giver*’ OR ‘care giving’ OR carer* OR communit* OR dyad* OR families OR family OR outpatient* OR patient* OR personal) NEAR/2 (continuit* OR coordinat* OR ‘co ordinat*’ OR mapping OR navigat* OR pathway* OR ‘path way*’ OR team* OR transition*)):ti,ab,kw	62,836
9	((biopsychosocial OR ‘bio psychosocial’ OR ‘bio psycho social’ OR crossdepartment* OR ‘cross department*’ OR crossdisciplin* OR ‘cross disciplin*’ OR crossprofession* OR ‘cross profession*’ OR interdepartment* OR ‘inter department*’ OR interdisciplin* OR ‘inter disciplin*’ OR interprofession* OR ‘inter profession*’ OR multidepartment* OR ‘multi department*’ OR multidisciplin* OR ‘multi disciplin*’ OR multimodal OR ‘multi modal’ OR multiprofession* OR ‘multi profession*’) NEAR/2 (approach* OR care OR case* OR collaboration* OR coordinat* OR ‘co ordinat*’ OR continuit* OR continuum* OR cooperat* OR facilitat* OR guide OR guides OR healthcare OR integrat* OR intervention* OR link* OR navigat* OR network* OR neurorehab* OR ‘neuro rehab*’ OR partnership* OR pathway* OR ‘path way*’ OR program* OR rehab* OR service* OR support* OR team* OR transition*)):ti,ab,kw	211,093
10	((app OR apps OR digital OR discharg* OR ehealth OR ‘e health’ OR internet OR mhealth OR ‘m health’ OR online OR remote* OR phone* OR smartphone* OR telephone*) NEAR/3 (followup* OR ‘follow* up’ OR ‘follow ups’ OR intervention OR interventions)):ti,ab,kw	53,590
11	(mobile NEAR/3 health* NEAR/3 (followup* OR ‘follow* up’ OR ‘follow ups’ OR intervention OR interventions)):ti,ab,kw	1439
12	#1 OR #2 OR #3 OR #4 OR #5 OR #6 OR #7 OR #8 OR #9 OR #10 OR #11	821,770
13	(‘daily life activity’/de OR ‘community care’/exp OR ‘facilities and services utilization’/de OR ‘health disparity’/de OR ‘health care access’/exp OR ‘health insurance’/exp OR ‘home care’/de OR ‘home visit’/de OR ‘hospital readmission’/de OR ‘medically uninsured’/de OR ‘needs assessment’/de OR ‘patient care planning’/exp OR ‘patient compliance’/exp OR ‘person centered care’/de OR ‘public-private partnership’/de OR ‘quality of life’/de OR ‘return to school’/exp OR ‘return to work’/de OR ‘self care’/exp OR ‘social care’/exp OR ‘visiting nursing service’/de OR ‘wellbeing’/exp)	2094,878
14	((reenter* OR ‘re enter*’ OR reentr* OR ‘re entr*’ OR return*) NEAR/2 (college* OR job OR jobs OR school* OR universit* OR work*)):ti,ab,kw	22,914
15	‘advance* directive*’:ti,ab,kw OR ‘advance* plan*’:ti,ab,kw OR ‘care goal*’:ti,ab,kw OR ‘care plan*’:ti,ab,kw OR ‘daily life’:ti,ab,kw OR ‘daily living’:ti,ab,kw OR financ*:ti,ab,kw OR food*:ti,ab,kw OR ‘functional status*’:ti,ab,kw OR ‘goals of care’:ti,ab,kw OR housing:ti,ab,kw OR hrqol:ti,ab,kw OR insur*:ti,ab,kw OR ‘living situation*’:ti,ab,kw OR malnourish*:ti,ab,kw OR ‘mal nourish*’:ti,ab,kw OR malnutrition:ti,ab,kw OR ‘mal nutrition’:ti,ab,kw OR medicaid:ti,ab,kw OR medicare*:ti,ab,kw OR monetar*:ti,ab,kw OR ‘need* assessment*’:ti,ab,kw OR psychosocial*:ti,ab,kw OR ‘psycho social*’:ti,ab,kw OR qol:ti,ab,kw OR ((quality NEAR/3 life):ti,ab,kw) OR readmission*:ti,ab,kw OR ‘re admission*’:ti,ab,kw OR readmit*:ti,ab,kw OR ‘re admit*’:ti,ab,kw OR reemploy*:ti,ab,kw OR ‘re employ*’:ti,ab,kw OR rehospital*:ti,ab,kw OR ‘re hospital*’:ti,ab,kw OR reintegrat*:ti,ab,kw OR ‘re integrat*’:ti,ab,kw OR ‘self care’:ti,ab,kw OR ‘self manag*’:ti,ab,kw OR snap:ti,ab,kw OR ‘supplemental nutrition’:ti,ab,kw OR underinsur*:ti,ab,kw OR unemploy*:ti,ab,kw OR unempower*:ti,ab,kw OR uninsur*:ti,ab,kw OR wic:ti,ab,kw	2559,105
16	((caregiv* OR ‘care giver*’ OR ‘care giving’ OR carer* OR communit* OR dyad* OR families OR family OR outpatient* OR participant* OR patient* OR person OR persons OR personali* OR population* OR proxies OR proxy OR public) NEAR/3 (access* OR adher* OR aid OR aide* OR assist* OR barrier* OR burden* OR centered* OR centred* OR comply OR complian* OR connect* OR employment OR educat* OR empower* OR enroll* OR experience OR experiences OR integrat* OR job OR jobs OR nonadher* OR noncomplian* OR partner* OR preference* OR recruit* OR support* OR workplace* OR ‘work place*’ OR workforce* OR ‘work force*’)):ti,ab,kw	1454,760
17	#13 OR #14 OR #15 OR #16	4643,600
18	‘central nervous system tumor’/exp OR ‘neuroepithelioma’/exp	472,570
19	‘central nervous system’/exp AND ‘neoplasm’/exp	201,538
20	astroblastoma*:ti,ab,kw OR astrocytoma*:ti,ab,kw OR atrt*:ti,ab,kw OR ‘atypical teratoid rhabdoid tumo*’:ti,ab,kw OR chondroma*:ti,ab,kw OR chondrosarcoma*:ti,ab,kw OR ependymoma*:ti,ab,kw OR esthesioneuroblastoma*:ti,ab,kw OR gangliocytoma*:ti,ab,kw OR ganglioglioma*:ti,ab,kw OR ganglioneuroblastoma*:ti,ab,kw OR glioma*:ti,ab,kw OR glioblastoma*:ti,ab,kw OR ‘glio blastoma*’:ti,ab,kw OR gliosarcoma*:ti,ab,kw OR ‘glio sarcoma*’:ti,ab,kw OR haemangiopericytoma*:ti,ab,kw OR ‘haemangio pericytoma*’:ti,ab,kw OR hemangiopericytoma*:ti,ab,kw OR ‘hemangio pericytoma*’:ti,ab,kw OR liponeurocytoma*:ti,ab,kw OR medulloblastoma*:ti,ab,kw OR ‘medullo blastoma*’:ti,ab,kw OR medulloepithelioma*:ti,ab,kw OR ‘medullo epithelioma*’:ti,ab,kw OR meningioma*:ti,ab,kw OR neuroblastoma*:ti,ab,kw OR ‘neuro blastoma*’:ti,ab,kw OR neurocytoma*:ti,ab,kw OR ‘neuro cytoma*’:ti,ab,kw OR neuroepithelioma*:ti,ab,kw OR ‘neuro epithelioma*’:ti,ab,kw OR neuroleukemi*:ti,ab,kw OR ‘neuro leukemi*’:ti,ab,kw OR neuroleukaemi*:ti,ab,kw OR ‘neuro leukaemi*’:ti,ab,kw OR neurooncolog*:ti,ab,kw OR ‘neuro oncolog*’:ti,ab,kw OR oligoastrocytoma*:ti,ab,kw OR oligodendrocytoma*:ti,ab,kw OR ‘oligo dendrocytoma*’:ti,ab,kw OR ‘oligodendro cytoma*’:ti,ab,kw OR ‘oligo dendro cytoma*’:ti,ab,kw OR oligodendroglioma*:ti,ab,kw OR ‘oligo dendroglioma*’:ti,ab,kw OR pcnsl*:ti,ab,kw OR pineoblastoma*:ti,ab,kw OR ‘pineoblastoma*’:ti,ab,kw OR pineocytoma*:ti,ab,kw OR ‘pineocytoma*’:ti,ab,kw OR rhabdomyosarcoma*:ti,ab,kw OR ‘rhabdo myosarcoma*’:ti,ab,kw OR ‘rhabdomyo sarcoma*’:ti,ab,kw OR subependymoma*:ti,ab,kw OR teratoid:ti,ab,kw OR xanthoastrocytoma*:ti,ab,kw	315,280
21	((brain* OR cns OR ‘central nervous system*’ OR cerebr* OR cranial* OR cranium* OR dura OR dural OR extradura* OR glia* OR intracrani* OR ‘intra crani*’ OR intradura* OR intramedul* OR ‘intra medul*’ OR leptomenin* OR ‘lepto menin*’ OR mater OR mening* OR meninx* OR ‘optic nerve*’ OR paramenin* OR paranasal* OR ‘para nasal*’ OR pineal* OR pituitar* OR spine OR spines OR spinal) NEAR/3 (blastoma* OR cancer* OR carcinoma* OR leukemi* OR leukaemi* OR lymphoma* OR malignan* OR metasta* OR neoplasm* OR oncolog* OR sarcoma* OR tumor OR tumour OR tumors OR tumours)):ti,ab,kw	351,959
22	#18 OR #19 OR #20 OR #21	721,651
23	#12 AND #17 AND #22	4130
24	#23 AND [english]/lim AND [1990–2024]/py	3976

APA PsycInfo (Ovid) Search, 9/26/24

1806 to September 2024 Week 3

220 Results

**Table:**

1	exp aftercare/ or biopsychosocial approach/ or exp case management/ or “continuum of care”/ or discharge planning/ or exp home care/ or home care personnel/ or home visiting programs/ or integrated services/ or interdisciplinary treatment approach/ or exp multimodal treatment approach/ or posttreatment followup/	109,137
2	(adherence support* or aftercare* or after-care* or assistance program* or coordinated approach* or navigator* or social prescri* or support* discharg*).ti,ab,id.	8639
3	((case or care) adj4 (comanage* or co-manage* or embed* or integrat* or management or manager* or map* or model or models or network* or pattern* or partnership* or transition*)).ti,ab,id.	55,536
4	((admiral or coordinat* or co-ordinat* or navigat* or phased or pivot) adj1 nurs*).ti,ab,id.	250
5	((home* or house*) adj2 (call* or visit*)).ti,ab,id.	6723
6	((advoca* or centrali* or collaborat* or coordinat* or co-ordinat* or continuit* or cooperat* or navigat* or pathway* or path-way* or shared) adj4 (care or consult* or health* or intervention or interventions or partnership* or program* or service*)).ti,ab,id.	52,270
7	(comprehensive* adj4 (care or consult* or followup* or follow* up or follow ups or intervention or interventions or management or network* or partnership* or program* or service*)).ti,ab,id.	16,636
8	((caregiv* or care giver* or care giving or carer* or communit* or dyad* or families or family or outpatient* or patient* or personal) adj2 (continuit* or coordinat* or co-ordinat* or mapping or navigat* or pathway* or path-way* or team* or transition*)).ti,ab,id.	11,610
9	((biopsychosocial or bio-psychosocial or bio-psycho-social or crossdepartment* or cross-department* or crossdisciplin* or cross-disciplin* or crossprofession* or cross-profession* or interdepartment* or inter-department* or interdisciplin* or inter-disciplin* or interprofession* or inter-profession* or multidepartment* or multi-department* or multidisciplin* or multi-disciplin* or multimodal or multi-modal or multiprofession* or multi-profession*) adj2 (approach* or care or case* or collaboration* or coordinat* or co-ordinat* or continuit* or continuum* or cooperat* or facilitat* or guide or guides or healthcare or integrat* or intervention* or link* or navigat* or network* or neurorehab* or neuro-rehab* or partnership* or pathway* or path-way* or program* or rehab* or service* or support* or team* or transition*)).ti,ab,id.	35,218
10	((app or apps or digital or discharg* or ehealth or e-health or internet or mhealth or m-health or (mobile adj3 health*) or online or remote* or phone* or smartphone* or telephone*) adj3 (followup* or follow* up or follow ups or intervention or interventions)).ti,ab,id.	12,512
11	1 or 2 or 3 or 4 or 5 or 6 or 7 or 8 or 9 or 10	257,042
12	community health/ or community resources/ or exp community services/ or community welfare services/ or functional status/ or exp health care access/ or exp health care utilization/ or exp health disparities/ or exp insurance/ or medicaid/ or medicare/ or needs assessment/ or patient centered care/ or exp “quality of life”/ or reemployment/ or reentry students/ or self-care/ or exp self-care skills/ or exp self-management/ or exp social integration/ or social security/ or social services/ or exp social support/ or treatment compliance/ or exp treatment planning/ or “underinsured (health insurance)”/ or “uninsured (health insurance)”/ or “welfare services (government)”/	280,853
13	((reenter* or re-enter* or reentr* or re-entr* or return*) adj2 (college* or job or jobs or school* or universit* or work*)).ti,ab,id.	6076
14	(advance* directive* or advance* plan* or care goal* or care plan* or daily life or daily living or financ* or food* or functional status* or “goals of care” or housing or HRQOL or insur* or living situation* or malnourish* or mal-nourish* or malnutrition or mal-nutrition or Medicaid or Medicare* or monetar* or need* assessment* or psychosocial* or psycho-social* or QOL or (quality adj3 life) or readmission* or re-admission* or readmit* or re-admit* or reemploy* or re-employ* or rehospital* or re-hospital* or reintegrat* or re-integrat* or self care or self manag* or SNAP or supplemental nutrition or underinsur* or unemploy* or unempower* or uninsur* or WIC).ti,ab,id.	510,312
15	((caregiv* or care giver* or care giving or carer* or communit* or dyad* or families or family or outpatient* or participant* or patient* or person or persons or personali* or population* or proxies or proxy or public) adj3 (access* or adher* or aid or aide* or assist* or barrier* or burden* or centered* or centred* or comply or complian* or connect* or employment or educat* or empower* or enroll* or experience or experiences or integrat* or job or jobs or nonadher* or noncomplian* or partner* or preference* or recruit* or support* or workplace* or work place* or workforce* or work force*)).ti,ab,id.	336,698
16	12 or 13 or 14 or 15	904,848
17	exp nervous system neoplasms/	5109
18	exp central nervous system/ and exp neoplasms/	2771
19	(astroblastoma* or astrocytoma* or ATRT* or atypical teratoid rhabdoid tumo* or chondroma* or chondrosarcoma* or ependymoma* or esthesioneuroblastoma* or gangliocytoma* or ganglioglioma* or ganglioneuroblastoma* or glioma* or glioblastoma* or glio-blastoma* or gliosarcoma* or glio-sarcoma* or haemangiopericytoma* or haemangio-pericytoma* or hemangiopericytoma* or hemangio-pericytoma* or liponeurocytoma* or medulloblastoma* or medullo-blastoma* or medulloepithelioma* or medullo-epithelioma* or meningioma* or neuroblastoma* or neuro-blastoma* or neurocytoma* or neuro-cytoma* or neuroepithelioma* or neuro-epithelioma* or neuroleukemi* or neuro-leukemi* or neuroleukaemi* or neuro-leukaemi* or neurooncolog* or neuro-oncolog* or oligoastrocytoma* or oligodendrocytoma* or oligo-dendrocytoma* or oligodendro-cytoma* or oligo-dendro-cytoma* or oligodendroglioma* or oligo-dendroglioma* or PCNSL* or pineoblastoma* or pineoblastoma* or pineocytoma* or pineocytoma* or rhabdomyosarcoma* or rhabdo-myosarcoma* or rhabdomyo-sarcoma* or subependymoma* or teratoid or xanthoastrocytoma*).ti,ab,id.	6908
20	((brain* or CNS or central nervous system* or cerebr* or cranial* or cranium* or dura or dural or extradura* or glia* or intracrani* or intra-crani* or intradura* or intramedul* or intra-medul* or leptomenin* or lepto-menin* or mater or mening* or meninx* or optic nerve* or paramenin* or paranasal* or para-nasal* or pineal* or pituitar* or spine or spines or spinal) adj3 (blastoma* or cancer* or carcinoma* or leukemi* or leukaemi* or lymphoma* or malignan* or metasta* or neoplasm* or oncolog* or sarcoma* or tumor or tumour or tumors or tumours)).ti,ab,id.	7088
21	17 or 18 or 19 or 20	13,039
22	11 and 16 and 21	243
23	limit 22 to (english language and yr=“1990 -Current”)	220

Cochrane Central (Wiley) Search, 9/26/24

Issue 8 of 12, August 2024

278 Results

**Table:**

1	((adherence NEXT support*) OR aftercare* OR after-care* OR (assistance NEXT program*) OR (coordinated NEXT approach*) OR navigator* OR (social NEXT prescri*) OR (support* NEXT discharg*)):ti,ab,kw	4823
2	((case OR care) NEAR/4 (comanage* OR co-manage* OR embed* OR integrat* OR management OR manager* OR map* OR model OR models OR network* OR pattern* OR partnership* OR transition*)):ti,ab,kw	24,124
3	((admiral OR coordinat* OR co-ordinat* OR navigat* OR phased OR pivot) NEAR/1 (nurs*)):ti,ab,kw	402
4	((home* OR house*) NEAR/2 (call* OR visit*)):ti,ab,kw	6116
5	((advoca* OR centrali* OR collaborat* OR coordinat* OR co-ordinat* OR continuit* OR cooperat* OR navigat* OR pathway* OR path-way* OR shared) NEAR/4 (care OR consult* OR health* OR intervention OR interventions OR partnership* OR program* OR service*)):ti,ab,kw	15,102
6	((comprehensive*) NEAR/4 (care OR consult* OR followup* OR (follow* NEXT up) OR “follow ups” OR intervention OR interventions OR management OR network* OR partnership* OR program* OR service*)):ti,ab,kw	6822
7	((caregiv* OR (care NEXT giver*) OR “care giving” OR carer* OR communit* OR dyad* OR families OR family OR outpatient* OR patient* OR personal) NEAR/2 (continuit* OR coordinat* OR co-ordinat* OR mapping OR navigat* OR pathway* OR path-way* OR team* OR transition*)):ti,ab,kw	8521
8	((biopsychosocial OR bio-psychosocial OR bio-psycho-social OR crossdepartment* OR cross-department* OR crossdisciplin* OR cross-disciplin* OR crossprofession* OR cross-profession* OR interdepartment* OR inter-department* OR interdisciplin* OR inter-disciplin* OR interprofession* OR inter-profession* OR multidepartment* OR multi-department* OR multidisciplin* OR multi-disciplin* OR multimodal OR multi-modal OR multiprofession* OR multi-profession*) NEAR/2 (approach* OR care OR case* OR collaboration* OR coordinat* OR co-ordinat* OR continuit* OR continuum* OR cooperat* OR facilitat* OR guide OR guides OR healthcare OR integrat* OR intervention* OR link* OR navigat* OR network* OR neurorehab* OR neuro-rehab* OR partnership* OR pathway* OR path-way* OR program* OR rehab* OR service* OR support* OR team* OR transition*)):ti,ab,kw	10,740
9	((app OR apps OR digital OR discharg* OR ehealth OR e-health OR internet OR mhealth OR m-health OR (mobile NEAR/3 health*) OR online OR remote* OR phone* OR smartphone* OR telephone*) NEAR/3 (followup* OR (follow* NEXT “up”) OR “follow ups” OR intervention OR interventions)):ti,ab,kw	25,199
10	#1 OR #2 OR #3 OR #4 OR #5 OR #6 OR #7 OR #8 OR #9	82,878
11	((reenter* OR re-enter* OR reentr* OR re-entr* OR return*) NEAR/2 (college* OR job OR jobs OR school* OR universit* OR work*)):ti,ab,kw	3354
12	((advance* NEXT directive*) OR (advance* NEXT plan*) OR (care NEXT goal*) OR (care NEXT plan*) OR “daily life” OR “daily living” OR financ* OR food* OR (“functional” NEXT status*) OR “goals of care” OR housing OR HRQOL OR insur* OR (“living” NEXT situation*) OR malnourish* OR mal-nourish* OR malnutrition OR mal-nutrition OR Medicaid OR Medicare* OR monetar* OR (need* NEXT assessment*) OR psychosocial* OR psycho-social* OR QOL OR (quality NEAR/3 life) OR readmission* OR re-admission* OR readmit* OR re-admit* OR reemploy* OR re-employ* OR rehospital* OR re-hospital* OR reintegrat* OR re-integrat* OR “self care” OR (“self” NEXT manag*) OR SNAP OR “supplemental nutrition” OR underinsur* OR unemploy* OR unempower* OR uninsur* OR WIC):ti,ab,kw	325,161
13	((caregiv* OR (“care” NEXT giver*) OR “care giving” OR carer* OR communit* OR dyad* OR families OR family OR outpatient* OR participant* OR patient* OR person OR persons OR personali* OR population* OR proxies OR proxy OR public) NEAR/3 (access* OR adher* OR aid OR aide* OR assist* OR barrier* OR burden* OR centered* OR centred* OR comply OR complian* OR connect* OR employment OR educat* OR empower* OR enroll* OR experience OR experiences OR integrat* OR job OR jobs OR nonadher* OR noncomplian* OR partner* OR preference* OR recruit* OR support* OR workplace* OR (“work” NEXT place*) OR workforce* OR (“work” NEXT force*))):ti,ab,kw	244,645
14	#11 OR #12 OR #13	498,264
15	(astroblastoma* OR astrocytoma* OR ATRT* OR (“atypical teratoid rhabdoid” NEXT tumo*) OR chondroma* OR chondrosarcoma* OR ependymoma* OR esthesioneuroblastoma* OR gangliocytoma* OR ganglioglioma* OR ganglioneuroblastoma* OR glioma* OR glioblastoma* OR glio-blastoma* OR gliosarcoma* OR glio-sarcoma* OR haemangiopericytoma* OR haemangio-pericytoma* OR hemangiopericytoma* OR hemangio-pericytoma* OR liponeurocytoma* OR medulloblastoma* OR medullo-blastoma* OR medulloepithelioma* OR medullo-epithelioma* OR meningioma* OR neuroblastoma* OR neuro-blastoma* OR neurocytoma* OR neuro-cytoma* OR neuroepithelioma* OR neuro-epithelioma* OR neuroleukemi* OR neuro-leukemi* OR neuroleukaemi* OR neuro-leukaemi* OR neurooncolog* OR neuro-oncolog* OR oligoastrocytoma* OR oligodendrocytoma* OR oligo-dendrocytoma* OR oligodendro-cytoma* OR oligo-dendro-cytoma* OR oligodendroglioma* OR oligo-dendroglioma* OR PCNSL* OR pineoblastoma* OR pineoblastoma* OR pineocytoma* OR pineocytoma* OR rhabdomyosarcoma* OR rhabdo-myosarcoma* OR rhabdomyo-sarcoma* OR subependymoma* OR teratoid OR xanthoastrocytoma*):ti,ab,kw	6378
16	((brain* OR CNS OR (“central nervous” NEXT system*) OR cerebr* OR cranial* OR cranium* OR dura OR dural OR extradura* OR glia* OR intracrani* OR intra-crani* OR intradura* OR intramedul* OR intra-medul* OR leptomenin* OR lepto-menin* OR mater OR mening* OR meninx* OR (optic NEXT nerve*) OR paramenin* OR paranasal* OR para-nasal* OR pineal* OR pituitar* OR spine OR spines OR spinal) NEAR/3 (blastoma* OR cancer* OR carcinoma* OR leukemi* OR leukaemi* OR lymphoma* OR malignan* OR metasta* OR neoplasm* OR oncolog* OR sarcoma* OR tumor OR tumour OR tumors OR tumours)):ti,ab,kw	11,197
17	#15 OR #16	14,796
18	#10 AND #14 AND #17	287
	with Publication Year from 1990 to 2024, in Trials	280
	English	278

Duplicate Removal Results Appendix 2

**Table:**

Database	Before Removing Duplicates	After Removing Duplicates	Number of Duplicates
Medline ALL (Ovid)	1813	1811	2
Embase (Elsevier)	3976	2877	1099
PsycInfo	220	113	107
Cochrane Central (Wiley)	278	162	116
**Total:**	**6287**	**4963**	**1324**

## Appendix 2. Data extraction methodology

**Table:**

Category	Description
**Citation**	Article citation details (author(s) and year published)
**Study name**	Title of study including name of patient navigation program or intervention
**Geographic Location**	State (if applicable) and nation of hospital / facility where program is conducted.
**Study Design**	Type of study: Case report, case-control study, cohort study, RCT, Non-RCT, longitudinal study or mixed methods.
**Inclusion Criteria**	
**Population / Condition**	Characteristics of the participants: age, diagnosis status, socio-economic status, and / or health insurance coverage.
**Types of Tumors Targeted**	The cancer types seen in the patient population
**Team Composition**	The team composition of conducting the patient navigation program.
**Navigator Title**	The title of the navigator on the team conducting the patient navigation program.
**Program Delivery Format**	Mode of distribution i.e., online, telephone, or in person. Care coordinator, interdisciplinary team, or physician led.
**Outcomes Studied**	Specific health outcome, disparity, number of resources utilized, or needs targeted by the intervention.
**Patient Experience**	The results of the navigation program include significant and non-significant impacts and patient outcomes and satisfaction.
**Clinician / Care team experience**	The clinician’s perspective on the program includes limitations either to the efficacy of or to the article’s study design and future steps.
**Needs Expressed by Patient**	Reported patient needs either through

## Appendix 3. Study identification and participant demographics

**Table:**

Author, year	Geographic location	Title	Study Design	Inclusion criteria	Population / Condition	Population Size	Types of Tumors Targeted
Hong et al., 2024 [30]	New South Wales, Australia	The impact of brain cancer care coordinators on healthcare utilization and outcomes in patients with glioblastoma	Retrospective cohort study	Patients diagnosed with a glioblastoma at the Hunter New England Local Health District, Australia within the timeframe of October 2012 to December 2019. Age of 18 or older.	Patients diagnosed with glioblastoma	187 Patients	Glioblastoma
Fleege et al., 2022 [38]	Michigan, United States	IMPACT the Brain: A Team-Based Approach to Management of Metastatic Breast Cancer with CNS Metastases	Prospective cohort study	Patients with metastatic breast cancer (MBC) and CNS metastases	Patients with metastatic breast cancer (MBC) and CNS metastases to the brain and the leptomeninges.	60 Patients	Breast cancer and brain / leptomeningeal metastases
Sadigh et al., 2019 [39]	Georgia, United States	Pilot Feasibility Study of an Oncology Financial Navigation Program in Brain Cancer Patients	Prospective single-arm cohort longitudinal observational study	Adult patients with newly diagnosed brain cancer over the age of 18; with a record of a plan between patient and physician to undergo any surgery, chemotherapy, or radiation therapy.	Patients with newly diagnosed brain tumors or brain metastases	12 Patients	Primary brain tumor (non-specified); Brain tumor metastases (non-specified)
Miller et al., 2018 [31]	Pennsylvania, United States	Neuro-Oncology Nurse Navigation: Developing the role for a unique patient population	Non-study Program	Brain tumor patient	Patients with brain tumors	–	Brain tumors
Bailey et al., 2015 [32]	Sydney, Australia	Australian Experience of Neuro-Oncology Care Coordination: A Conversation	Non-study Program	Patients with primary brain tumors and spinal tumors.	Patients with primary brain tumors and spinal tumors.	–	Primary brain tumors and spinal tumors
Akingbade et al., 2024 [43]	Ontario, Canada	Enhancing the patient journey to clinical trial enrollment with navigation to optimize accrual: A pilot study for a pragmatic multicenter, stepped wedge, cluster randomized controlled trial (The CTN Pilot Trial)	Prospective cohort study	Canadian patients with various cancer diagnoses referred to the CTN program between March 2019 to January 2024.	Canadian patients with various cancer diagnoses.	373 Patients	Variety of cancers including lung, lymphoma, pancreatic brain, and prostate cancers.
Bruun-Pedersen et al., 2023 [33]	Copenhagen, Denmark	The Value of a Nurse Care Coordinator Working with Young Brain Tumor Patients in the Neurosurgical Outpatient Clinic	Non-study Program	Young patients between 18–35 years old with brain cancer tumors.	Young patients between 18–35 years old with brain cancer tumors.	20 Patients	Brain cancer tumors
Patel et al., 2022 [40]	California, United States	Effect of a Community Health Worker Intervention of Acute Care Use, Advance Care Planning, and Patient-Reported Outcomes Among Adults with Advanced Stages of Cancer: A Randomized Clinical Trial	Randomized Controlled Trial	Patients with newly diagnosed advanced-stage or recurrent solid and hematologic cancers from August 8, 2017, through November 30, 2021	Patients with newly diagnosed advanced-stage or recurrent solid and hematologic cancers	128 Participants	Newly diagnosed advanced stages or recurrent solid and hematologic cancers, patients with brain tumor reactions
Pentsova, 2020 [61]	New York, United States	A Comprehensive Model for Patients with Central Nervous System (CNS) Cancers: Results of New Approach	Prospective cohort study	CNS cancers	CNS Tumors, 79% high grade glioma	82 Patients	High-grade glioma/glioblastoma
Grogan et al., 2021 [42]	Michigan, United States	Evaluating specialty referral, research involvement, and PROs in a CNS-involved metastatic breast cancer care coordination program.	Prospective cohort study	Patients with metastatic breast cancer (MBC) that have central nervous system (CNS) metastases	Patients with metastatic breast cancer (MBC) that have central nervous system (CNS) metastases	43 Patients	Metastatic breast cancer (MBC) and Central Nervous System (CNS) metastases
Duflot-Boukobza et al., 2020 [34]	Villejuif, France	Intervention combining nurse navigators (NNs) and a mobile application vs standard of care (SOC) in neuro-oncology patients (pts) treated with oral anticancer agents (OAA): A subgroup analysis of CAPRI, a single-center, randomized phase III trial	Randomized controlled trial	Adult cancer patients treated with oral anticancer agents (OAA).	Glioblastoma patients receiving standard of care treatment	51 Patients	Glioblastoma
Mains et al., 2020 [35]	Ohio, United States	Postoperative Care Coordination for Acoustic Neuroma Patients: Improving Patient Satisfaction	Non-study Program	Acoustic Neuroma patients	Acoustic Neuroma patients	60 Patients	Acoustic Neuroma
Hamm et al., 2020 [52]	Canada	Clinical trials navigator: Patient-centered access to clinical trials	Prospective cohort study	Patients diagnosed with any cancer aged 18 or older	Cancer patients aged 18 or older	96 Patients	All tumor types included
Fernandez et al., 2019 [44]	Leeds, United Kingdom	The Impact of the Neuro-Oncology Research Radiographer Role in the Effective Facilitation of an Advanced Imaging Study in Glioblastoma	Prospective cohort study	Brain Tumor Patients	Brain Tumor Patients	12 Participants	Glioblastoma patients
Philip et al., 2017 [62]	Melbourne, Australia	I-CoPE: A Pilot Study of an Innovative Model of Supportive and Palliative Care for Patients with High Grade Glioma and their Family Carers	Prospective cohort study	Patients newly diagnosed with high grade glioma and their family carer	Patients newly diagnosed with High Grade Glioma (HGG)	32 Patients	High Grade Glioma (HGG)
Nixon et al., 2017 [36]	Newcastle, Australia	Care coordination: The experience of a brain cancer care coordinator in Australia	Non-study Program	Patients with Brain Cancer	Patients with Brain Cancer	–	Patients with Brain Cancer
Nghiemphu et al., 2017 [45]	California, United States	Improving care coordination for brain tumor patients through value-based care redesign of a virtual integrated practice unit	Prospective cohort study	Patients with brain tumor resections	Patients with brain tumor resections	202 Patients	Brain tumors
Lima et al., 2013 [49]	Ohio, United States	The Journey Begins: Implementation of a Novel Survivorship Care Delivery Model at The Time of Diagnosis for Patients with a Primary Brain Tumor	Non-study Program	Newly diagnosed primary brain tumor patients	Primary brain tumor patients in neuro-oncology clinics at time of diagnosis	–	Brain Cancer
Legge et al., 2013 [53]	Melbourne, Australia	Capitalizing on a Unique Opportunity: Establishing A Sustainable Brain Tumor Support Service in Melbourne, Australia	Non-study Program	Brain tumor patients and their families.	Brain Tumor Patients	–	Brain Cancers
Shepherd et al., 2012 [46]	Edinburgh, Scotland	Navigation’ for high grade brain tumor patients. A longitudinal qualitative evaluation of a shared decision-making intervention	Non-study Program	Patients diagnosed with a high-grade glioma attending the Edinburgh Centre for Neuro-oncology	Patients (n = 20) attending the Edinburgh Neuro-oncology Centre diagnosed with a High Grade brain tumor	26 Patients	High Grade brain tumor
Robinson et al., 2011 [37]	Sydney, Australia	Evaluating the impact of a neuro-oncology nurse coordinator in Southwest Sydney	Non-study Program	Patients with primary brain tumors	Patients with primary brain tumors	129 Patients	Primary brain tumors: high-grade gliomas, (HGGs; 70%), low-grade gliomas (LGG; 14%), and benign brain tumors (BBTs; 16%).
Guadagnolo et al., 2011 [50]	South Dakota, United States	Patient navigation for American Indians undergoing cancer treatment: utilization and impact on care delivery in a regional health care center	Prospective cohort study	Diagnosis of malignancy, Included American Indians from Oglala Sioux Tribe, Cheyenne River Sioux Tribe, Rosebud Sioux Tribe, and the Rapid City AI population	American Indians in the Rapid City, South Dakota area	332 Patients	Breast, Lung, Prostate, Colorectal, Head and neck, Gastric, Pancreas, hepatocellular, Sarcoma/Skin, Brain, and Thyroid Cancer

## Appendix 4. Patient navigation program team composition, and delivery format

**Table:**

Author, year	Title	Team composition	Navigator Title	Program Delivery Format
Hong et al., 2024	The impact of brain cancer care coordinators on healthcare utilization and outcomes in patients with glioblastoma	Brain Cancer Care Coordinators (BCCCs)	Brain Cancer Care Coordinators (BCCCs)	Special nurses, BCCCs, aid through in-person and phone call including comprehensive support of patients with diagnosis of primary brain tumors and their families through diagnosis, treatment, clinical trials, symptom management, follow-up in the community and end of life care.
Fleege et al., 2022	IMPACT the Brain: A Team-Based Approach to Management of Metastatic Breast Cancer with CNS Metastases	Breast medical oncology, radiation oncology, neurosurgery, neuro-oncology, palliative care, genetics, rehabilitation psychology/neuropsychology, and physical medicine and rehabilitation (PM&R).	Program Coordinator	The program coordinator completed an intake screening form designed specifically for this program with each patient via telephone. The coordinator provided navigation, education, specialty referral, and clinical trial screening. Patient-reported outcomes and caregiver burden assessments were collected. Patients voluntarily completed a questionnaire for patient-reported outcomes of the patient’s overall physical and behavioral functions after enrollment.
Sadigh et al., 2019	Pilot Feasibility Study of an Oncology Financial Navigation Program in Brain Cancer Patients	Study coordinator, Patient Advocate Foundation (PAF) Member, PAF case managers	PAF Case manager	Baseline survey and follow-up surveys at 3, 6, and 9 months after enrollment during follow-up clinic visits (paper survey) or on the telephone. The surveys included questions to assess financial toxicity, financial coping mechanisms, and care non-adherence because of cost of care. Patients who completed a baseline survey were invited to join a six-month financial navigation program. Case managers contacted participants monthly for six months.
Miller et al., 2018	Neuro-Oncology Nurse Navigation: Developing the role for a unique patient population	Neuro-oncology nurse navigator	Neuro-oncology nurse navigator	Triaging, coordinating appointments, communication with providers, education, supportive referrals (i.e., social work), coordination of transportation needs.
Bailey et al., 2015	Australian Experience of Neuro-Oncology Care Coordination: A Conversation	Neuro-oncology care coordinator, patient, caregivers, healthcare professionals	Neuro-oncology care coordinator	Patient-centered care delivery, increased supportive services for patients and caregivers, patient access to community and hospital resources
Akingbade et al., 2024	Enhancing the patient journey to clinical trial enrollment with navigation to optimize accrual: A pilot study for a pragmatic multicenter, stepped wedge, cluster randomized controlled trial (The CTN Pilot Trial)	Three non-medical navigators and physician-oncologist	Clinical Trial Navigator	Physician referral, non-medical navigator review of medical information, and search for eligible clinical trials in five different clinical trial search engines, and secondary physician review of trials.
Bruun-Pedersen et al., 2023	The Value of a Nurse Care Coordinator Working with Young Brain Tumor Patients in the Neurosurgical Outpatient Clinic	Nurse Care Coordinator, Neurosurgical Outpatient clinic, young patients, relatives of patients	Nurse care Navigator	Regular weekly phone calls and monthly conversations or personal meetings in the Outpatient Clinic discussing diagnosis, navigating barriers to rehabilitation and discussing solutions with external parties on behalf of the patient.
Patel et al., 2022	Effect of a Community Health Worker Intervention of Acute Care Use, Advance Care Planning, and Patient-Reported Outcomes Among Adults with Advanced Stages of Cancer- A Randomized Clinical Trial	–	Community Health Worker Intervention Program	One week after the first oncology appointment or disease recurrence. Twice monthly over 6 months. 3 telephone segments
Pentsova et al., 2020	A Comprehensive Model for Patients with Central Nervous System (CNS) Cancers: Final Results of New Approach	Neurologist, nurse, physiatrist, physical therapist, social worker, case manager, dietitian, and chaplain	Neurological Multidisciplinary Care Clinic (MdCC)	Clinic-based 3 h-visit, providers rotated in to see patients and their caregivers.
Grogan et al., 2021	Evaluating specialty referral, research involvement, and PROs in a CNS-involved metastatic breast cancer care coordination program.	Program Coordinator, breast medical oncology, breast cancer genetics, radiation oncology, neurosurgery, neuro-oncology, physical medicine and rehabilitation (PM&R), neuropsychology, and palliative care	Program Coordinator	Navigation, education, specialty referral, clinical trial screening, assessment of patient-reported outcomes (PROs), creation of expedited referrals specific to each participant, and use of the PROMIS Cancer Function Brief 3D Profile, Short Form Zarit Burden Interview (ZBI-12), MD Anderson Symptom Inventory Brain Tumor (MDASI), and a screen of the burden faced by caregivers.
Duflot-Boukobza et al., 2020	Intervention combining nurse navigators (NNs) and a mobile application vs standard of care (SOC) in neuro-oncology patients (pts) treated with oral anticancer agents (OAA): A subgroup analysis of CAPRI, a single-center, randomized phase III trial	Nurse Navigator	Nurse Navigator	Nurse Navigator mediated phone follow-ups to manage symptoms and assess toxicities, adherence and supportive care needs and mobile application for communication with NN intervention lasting six months.
Mains et al., 2020	Postoperative Care Coordination for Acoustic Neuroma Patients: Improving Patient Satisfaction	Nurse Navigator, and disciplines including neurosurgery, otolaryngology, physical therapy and rehab, facial rehabilitation and vestibular therapy.	Nurse Navigator	Nurse Navigator assists patients in navigation of their preoperative and postoperative care through providing written recommendations and guidance in the form of a booklet following acoustic neuroma resection.
Hamm et al., 2020	Clinical trials navigator: Patient-centered access to clinical trials	Clinical Trials Navigator (CTN)	Clinical Trials Navigator (CTN)	Using patient demographic and health information to determine eligibility for clinical trial options.
Fernandez et al., 2019	The Impact of the Neuro-Oncology Research Radiographer Role in the Effective Facilitation of an Advanced Imaging Study in Glioblastoma	Multi-disciplinary study team	Neuro-oncology research radiographer	Face-to-face interactions, email and telephone communication supporting patient recruitment, coordination of appointments and monitoring of patient progress.
Philip et al., 2017	I-CoPE: A Pilot Study of an Innovative Model of Supportive and Palliative Care for Patients with High Grade Glioma and their Family Carers	–	Cancer Care Coordinator	Patient Education, Regular Screening, Transfer of Information, and Family Caregiver Engagement at three transition points: post-diagnosis, post-diagnostic hospitalization, and post-radiotherapy
Nixon et al., 2017	Care coordination: The experience of a brain cancer care coordinator in Australia	Care Coordinator	Brain Cancer Care Coordinator (BCCC)	Provision of clinical advice, patient education, needs assessments and appropriate referrals.
Nghiemphu et al., 2017	Improving care coordination for brain tumor patients through value-based care redesign of a virtual integrated practice unit	–	Navigator Program	Post-surgical appointment scheduling. Patient education.
Lima et al., 2013	The Journey Begins: Implementation of a Novel Survivorship Care Delivery Model at The Time of Diagnosis for Patients with a Primary Brain Tumor	Nurse practitioner coordinator and primary neuro-oncologist	Nurse practitioner Coordinator	Survivorship visit including distress screenings, personalized notebook for patient symptom tracking, information regarding diagnosis, and available supportive care, and service and wellness initiatives like a walking challenge, with an after-visit summary, generated in the electronic medical record.
Legge et al., 2013	Capitalizing on a Unique Opportunity: Establishing A Sustainable Brain Tumor Support Service in Melbourne, Australia	–	–	Clinical education day for regional and metropolitan health professionals, day placement for regional health professionals with the Brain Tumor Support Service
Shepherd et al., 2012	Navigation’ for high grade brain tumor patients. A longitudinal qualitative evaluation of a shared decision-making intervention	Navigator, oncologist	Navigator	Navigators helped patients create a list of their questions for use in oncology consultation. patients were given an audio recording of their consultation (CD) and a written record of the key points discussed.
Robinson et al., 2011	Evaluating the impact of a neuro-oncology nurse coordinator in Southwest Sydney	Nurse Care Coordinator	Neuro-oncology nurse care coordinators (NOCCs)	Referral to nurse coordinator followed by outreach and needs assessment/psychosocial screening leading to allocation to supportive resources.
Guadagnolo et al., 2011	Patient navigation for American Indians undergoing cancer treatment: utilization and impact on care delivery in a regional health care center	Navigator, community research representatives	Walking Forward Program	In person, by telephone, or home visits to assist patients with navigating cancer therapy, obtaining medications, insurance issues, communicating with medical providers and travel and lodging logistics, and offer psycho-social support during treatment. Patient education materials were translated into the Lakota language.

## Appendix 5. Patient navigation program outcomes

**Table:**

Author, year	Title	Outcome Studied	Patient Experience	Clinician/Care Team Experience	Needs Expressed by Participants
Hong et al., 2024	The impact of brain cancer care coordinators on healthcare utilization and outcomes in patients with glioblastoma	Overall survival, health service resource use, odds of being admitted to hospital after the emergency presentation, and economic implications of BCCCs.	No significant differences in overall survival between groups. Reduction in number of ED presentations and admissions; 24% reduction in aggregate length of stay with the BCCC group. There is no statistically significant difference in mean patient costs, however the hospital may have saved over AUD $500,000 with BCCCs.	The exact nature of the interactions between the BCCC and the patients and its influence were unable to be accurately investigated. There is a possibility that not all interactions were recorded, and this may have led to documentation bias.	Patients who had more severe symptoms would have contacted the BCCC for medical advice.
Fleege et al., 2022	IMPACT the Brain: A Team-Based Approach to Management of Metastatic Breast Cancer with CNS Metastases	Feasibility of program implementation, frequency of visits the patients had with a sub-specialist after enrollment into the program and assessment of physical function, fatigue, and ability to participate in social roles and activities.	Faster time to first patient visit, enabled access to subspecialist care, and supported enrollment in clinical trials.	Addressing functional decline through increased PM&R referrals should be considered a standard of care in this population.	Patients expressed needs of addressing reduced physical function and increased fatigue causing at least moderate distress for half of the patients.
Sadigh et al., 2019	Pilot Feasibility Study of an Oncology Financial Navigation Program in Brain Cancer Patients	Patient – Case manager interactions, services provided, and financial aid secured. The study also measured changes in financial distress using COST scores and evaluated the program’s feasibility based on participation rates and types of support delivered. It also observed quality of life (self-reported) and health-insurance literacy.	At baseline, patients with brain cancer had a mean COST score significantly lower than the average score for solid tumor patients, indicating a higher risk of financial toxicity. 12 patients reported reduced spending due to treatment costs, 75% reported care nonadherence, and 58% experienced financial hardship, including borrowing money or using savings. There was no significant difference in the COST score of five patients who had follow-up scores at 3 months when compared with baseline. The total amount of debt relief provided through PAF case management work during the study period was $15,110.	The study faced challenges in recruitment and follow-up due to high patient mortality, cognitive decline, and caregiver dependence. Many patients were newly diagnosed and focused on treatment, making financial studies a lower priority. Despite this, the study demonstrated that centralized financial navigation is feasible when patients are engaged. Future efforts should include greater team involvement, patient incentives, diverse recruitment strategies, and improved communication about the financial aspects of care.	Debt crisis or cost of living, disability issues, employment issues, insurance issues, medical decision-making issues, and psychosocial support
Miller et al., 2018	Neuro-Oncology Nurse Navigation: Developing the role for a unique patient population	The study focused on navigation referrals, feasibility in recruiting patients for the program, increase in coordinated treatments for patients with brain metastases, facilitating communication with providers and facilities. Navigation referrals have increased drastically from 62 patients in 2015 to 408 patients in 2017. Patients starting radiation therapy within 35 days rose from 43%−60%.	Referrals come from newly diagnosed patients and established patients needing assistance in triaging, coordinating appointments, communication with providers, or referrals. Successful patient feedback and increase in patient volume. Navigation referrals have increased drastically from 62 patients in 2015 to 408 patients in 2017. Patients starting radiation therapy within 35 days rose from 43%−60%.	Successful feedback from providers. The ability of the neuro-oncology nurse navigator to bridge the specialties and transitions in care to create timely access to treatment is powerful demonstration of the role.	Newly diagnosed patients and established patients show needs, such as triaging, coordinating, appointments, communication with providers, education, and specialty providers or supportive care referrals.
Bailey et al., 2015	Australian Experience of Neuro-Oncology Care Coordination: A Conversation	Access to health services and supportive services for patients and their caregivers.	Patients and caregivers reported increased emotional support and resource access due to nurse navigators	NOCC services remain in their infancy, but a commitment should be made to establish the NOCC role on a statewide level, with the aim of offering this service nationally.	Neurocognitive burden, need for consistent information regarding diagnosis, and goal setting.
Akingbade et al., 2024	Enhancing the patient journey to clinical trial enrollment with navigation to optimize accrual: A pilot study for a pragmatic multicenter, stepped wedge, cluster randomized controlled trial (The CTN Pilot Trial)	Association between the type of cancer and the success of referral to a trial. Improvement of clinical trial accrual.	A fifth of patients who were referred to a clinical trial were enrolled and an increase in survival of referred patients from the last analysis from 3.0 months to 5.3 months.	Current changes to the program to improve these metrics and to improve scope of implementation.	Navigation and enrollment in clinical trials.
Bruun-Pedersen et al., 2023	The Value of a Nurse Care Coordinator Working with Young Brain Tumor Patients in the Neurosurgical Outpatient Clinic	Measure the value of a nurse care coordinator that would assist from the time of diagnosis through treatment course, ensuring appropriate rehabilitation and connection with external professionals. Overall, there was increased comfort and value to young patients with brain tumors allowing them more comfort in the neurosurgical outpatient clinic.	Decreased anxiety, allowed sense of comfort and familiarity	Nurse coordinators endorse higher multidisciplinary workflow	Understanding disease and treatment courses, barriers in rehabilitation, caregiver needs.
Patel et al., 2022	Effect of a Community Health Worker Intervention of Acute Care Use, Advance Care Planning, and Patient-Reported Outcomes Among Adults with Advanced Stages of Cancer: A Randomized Clinical Trial	The primary outcome was acute care use. Secondary outcomes included advance care planning documentation, supportive care use, patient-reported outcomes, survival, and end-of-life care use.	Intervention participants had 62% lower risk of acute care use than the control within 6 months. At 12 months, intervention participants had 17% lower odds of acute care use 8 times the odds of advance care planning documentation, 4 times the odds of palliative care, nearly double the odds of hospice, and nearly double the odds of improved mental and emotional health from enrollment to 6 and 12 months post enrollment. There were no differences in the death. There were no differences between groups in the proportion of participants who highly rated their satisfaction or their overall health status at any time point.	Study was conducted at a single community clinic with a low number of Black participants, limiting generalizability. Challenges also included slow participant accrual, incomplete cancer staging, and a small number of deaths by 12-month follow-up, suggesting the need for longer observation.	Acute care, emergency visits, goals of care discussion, advance directive, supportive, palliative, and hospice care.
Pentsova, 2020	A Comprehensive Model for Patients with Central Nervous System (CNS) Cancers: Results of New Approach	Setting goals of care, assessing their support system assessment, and development of a safe patient-centered plan of care.	Patient and caregivers unmet needs were effectively identified and addressed within a single MdCC visit the majority of which were highly satisfied and would recommend MdCC to others.	The presence of several providers through MdCC allows for the assessment of patients / caregivers’ needs and coping skills in real time.	Prognosis/coping with cancer, more education on signing health care proxy forms and DNR.
Grogan et al., 2021	Evaluating specialty referral, research involvement, and PROs in a CNS-involved metastatic breast cancer care coordination program.	Patient enrollment into research programs for those with metastases. Involvement in studies that included interventional therapy and non-interventional means of therapy. Number of patient referrals to subspecialties and patient completion of program questionnaires.	56 additional referrals were made to 7 sub-specialties. The care coordination program improved patient access to care among various sub-specialties and improved research participation for an underrepresented group.	The study team noted that the care coordination program improved healthcare access for their target population of patients with MBC and CNS metastases. Their questionnaires were optional for completion, and the researchers suspect that their inadequate rates of completion may reflect the unique cognitive and socio-economic challenges faced by this demographic. This may necessitate further research.	Needs for referral to various specialties including radiation oncology (n = 9), PM&R (n = 9), neuro-oncology (n = 8), and neuropsychology (n = 8).
Duflot-Boukobza et al., 2020	Intervention combining nurse navigators (NNs) and a mobile application vs standard of care (SOC) in neuro-oncology patients (pts) treated with oral anticancer agents (OAA): A subgroup analysis of CAPRI, a single-center, randomized phase III trial	Relative Dose intensity (RDI), adherence to care, toxicity, response and survival, quality of life, patient experience, end-of-life support, and economic estimation of the use of healthcare resources.	The intervention group had a significantly higher RDI, fewer emergency visits and hospitalizations, and better patient experience and use of supportive care resources, with no difference in treatment-related toxicity.	Intervention became standard in institutions and currently aims for larger-scale investigations in neuro-oncology patients.	Oral anticancer agent related toxicities and needs for management.
Mains et al., 2020	Postoperative Care Coordination for Acoustic Neuroma Patients: Improving Patient Satisfaction	Impact of specialized care on the pre and postoperative outcomes of patients along with the improved patient satisfaction.	Specialized nurse navigation for patients with skull base tumors, specifically acoustic neuroma, is an essential part of improving patient satisfaction and outcomes.	–	unique pre and postoperative needs of patients with acoustic neuroma.
Hamm et al., 2020	Clinical trials navigator: Patient-centered access to clinical trials	To assess issues of clinical trial availability. Number of patients who requested CTN services provided with a report within 24 h and the amount who died before referral and the median time from the referral to the CTN to the patients’ deaths.	69% of patients successfully completed follow-up. Of that 69%, 25% were deceased before referral, 22% were found ineligible for a trial, and 21% were on trial or were in the process of achieving a workup for a trial.	Significant interest from both physicians and patients for this service was identified. Strategies are being developed to encourage earlier referrals to clinical trials would improve number of patients entering clinical trials	Trial availability due to barriers such as poor trial design, inappropriate endpoints, inappropriate inclusion/exclusion factors.
Fernandez et al., 2019	The Impact of the Neuro-Oncology Research Radiographer Role in the Effective Facilitation of an Advanced Imaging Study in Glioblastoma	Patient recruitment and number / type of coordinator - patient interaction, patient satisfaction/feedback, and progress. Optimal treatment and patient-centered care were prioritized.	6 patients recruited so far specialists have attended 28 scans. Over 40 face-to-face interactions occurred and 30 email communications were sent. Patients value a consistent point of contact through the study	Effective liaison between the radiotherapy and imaging departments has been crucial throughout the study.	Support recruitment, coordinate appointments and monitor patients’ progress during advanced imaging study in glioblastoma.
Philip et al., 2017	I-CoPE: A Pilot Study of an Innovative Model of Supportive and Palliative Care for Patients with High Grade Glioma and their Family Carers	I-CoPE was well-received among patients and caretakers. Acceptability, quality of life, support needs, information needs, and care preparation were measured and observed marked improvement after intervention.	Patients reported fewer information needs and improvements in quality of life following their diagnostic hospitalization which were not sustained following radiotherapy. Carers reported fewer unmet supportive care and information needs, improved quality of life, and increased preparedness to care.	Supportive care delivery was successful; the aim is to conduct formal randomized studies.	Patients’ Information and quality of life needs, and supportive needs from carers.
Nixon et al., 2017	Care coordination: The experience of a brain cancer care coordinator in Australia	Efficiency of transitions through care, reduction in patient distress, and compliance with care.	Reduced patient distress and increased compliance with care.	Aim to improve the overall wellbeing of patients.	Navigating debilitating deficits, difficulty accessing services, lack of information, adverse effects from treatment, reduced social support.
Nghiemphu et al., 2017	Improving care coordination for brain tumor patients through value-based care redesign of a virtual integrated practice unit	Number of post-surgical follow-up appointments scheduled within 1 week of surgery and patient satisfaction.	Patients reported higher HCAHPS in areas of patient coordination and satisfaction.	Aim to improve the program by implementing centralized access to care.	Scheduling follow-up appointments for treatment planning within a week of their surgery.
Lima et al., 2013	The Journey Begins: Implementation of a Novel Survivorship Care Delivery Model at The Time of Diagnosis for Patients with a Primary Brain Tumor	Care coordination seems to allow patients to track their symptoms and see any signals of distress to potentially work with nurses and care teams to help with the symptoms. The survivorship care model aims to maximize quality of life, facilitate care coordination, and connect patients with appropriate resources	Calendar used to track symptoms, medications, chemotherapy, distress, personalized education notebook	Challenges include lack of clinic space and time to accommodate multiple disciplines, distance travelled by patients. Future goals include development of clinical practice guidelines and expanded services for caregivers.	Quality of life needs: symptom and distress management and patient education needs. Assistance with resource navigation and
Legge et al., 2013	Capitalizing on a Unique Opportunity: Establishing A Sustainable Brain Tumor Support Service in Melbourne, Australia	Patient demographics, clinical characteristics of patients, symptom burden and temporality and future, positive readiness expectancy and interconnectedness. It also addresses service gaps and aims to bridge gaps between the private and public health systems.	Patients not on active treatment and without tumor recurrence reported higher levels of hope than their counterparts. high symptom burden and interference is associated with lower hope scores.	Future studies need to focus on this relationship to implement ways to improve one’s coping skills in dealing with diagnoses and treatment changes.	Psycho-social coping with diagnosis.
Shepherd et al., 2012	Navigation’ for high grade brain tumor patients. A longitudinal qualitative evaluation of a shared decision-making intervention	Patients that underwent navigation felt more informed and were better equipped for understanding discussions with their consulting physician.	Creating a question list afforded patients the clarity to understand speaking points during consultation. Some found listening to the CD hard but felt able to return to it when ready.	Further research on making navigator-mediated question list making as a routine practice.	Understanding communication from oncologist and remembering key points.
Robinson et al., 2011	Evaluating the impact of a neuro-oncology nurse coordinator in Southwest Sydney	Support for patients and caregivers throughout the treatment journey and increase efficiency in clinical care delivery.	Large majority agreed or strongly agreed that the NOCC was essential to PBT patient care.	–	Various patient and caregiver psychosocial needs.
Guadagnolo et al., 2011	Patient navigation for American Indians undergoing cancer treatment: utilization and impact on care delivery in a regional health care center	Identify causes of disparities, navigator engagement, increase cancer screening and education, educate patients on clinical trials, and provide cultural-appropriate PN services throughout cancer treatment.	A decreasing proportion of patients needed help with care coordination/case management after the first visit but help with financial assistance increased after the first visit along with psycho-social support. A lower proportion of patients requested help with advocacy or cancer care education after their first visit. Fewer treatment interruptions for patients undergoing curative RT.	Better rapport with underserved populations and increase in clinical trial participation.	Obtaining medications, insurance issues, communicating with medical providers, and travel and lodging logistics.
